# **β**_3_-Adrenergic receptors regulate human brown/beige adipocyte lipolysis and thermogenesis

**DOI:** 10.1172/jci.insight.139160

**Published:** 2021-06-08

**Authors:** Cheryl Cero, Hannah J. Lea, Kenneth Y. Zhu, Farnaz Shamsi, Yu-Hua Tseng, Aaron M. Cypess

**Affiliations:** 1 Diabetes, Endocrinology, and Obesity Branch, National Institute of Diabetes and Digestive and Kidney Diseases (NIDDK), NIH, Bethesda, Maryland, USA.; 2 Integrative Physiology and Metabolism Section, Joslin Diabetes Center, Harvard Medical School, Boston, Massachusetts, USA.

**Keywords:** Cell Biology, Metabolism, Adipose tissue, G protein&ndash;coupled receptors, Molecular biology

## Abstract

β_3_-Adrenergic receptors (β_3_-ARs) are the predominant regulators of rodent brown adipose tissue (BAT) thermogenesis. However, in humans, the physiological relevance of BAT and β_3_-AR remains controversial. Herein, using primary human adipocytes from supraclavicular neck fat and immortalized brown/beige adipocytes from deep neck fat from 2 subjects, we demonstrate that the β_3_-AR plays a critical role in regulating lipolysis, glycolysis, and thermogenesis. Silencing of the β_3_-AR compromised genes essential for thermogenesis, fatty acid metabolism, and mitochondrial mass. Functionally, reduction of β_3_-AR lowered agonist-mediated increases in intracellular cAMP, lipolysis, and lipolysis-activated, uncoupling protein 1–mediated thermogenic capacity. Furthermore, mirabegron, a selective human β_3_-AR agonist, stimulated BAT lipolysis and thermogenesis, and both processes were lost after silencing β_3_-AR expression. This study highlights that β_3_-ARs in human brown/beige adipocytes are required to maintain multiple components of the lipolytic and thermogenic cellular machinery and that β_3_-AR agonists could be used to achieve metabolic benefit in humans.

## Introduction

Brown adipose tissue (BAT) is the principal thermogenic organ in mammals with the purpose of increasing energy expenditure in response to cold (nonshivering thermogenesis) or nutritional overload (diet-induced thermogenesis) (1, 2). In rodent models, BAT is primarily located in the interscapular region (iBAT), and its activation leads to improvement in obesity, glucose metabolism, and atherosclerosis (3, 4). The physiological roles of BAT in rodents have grown with the identification of various classes of mediators that affect at least liver and muscle lipid and glucose metabolism (5–8). Far less is known about the regulation and roles of human BAT thermogenesis. Originally believed to be only present in newborns, metabolically active BAT has been identified in adult humans via PET/CT imaging located mainly in supraclavicular fossa and axilla, followed by the mediastinal, paraspinal, perinephric, and supradrenal areas (9–13). Human BAT thermogenesis may have physiological relevance as there is an inverse correlation between active BAT and obesity (9, 14, 15). Notably, activation of BAT by cold exposure is associated with increased insulin sensitivity (16). Given these potential effects on glucose and lipid metabolism, investigating the physiological and metabolic relevance of human BAT is of great interest.

Rodent iBAT is composed primarily of “classical” brown adipocytes enriched with mitochondria and the thermogenic protein uncoupling protein 1 (UCP1). In contrast, developmental and molecular characterization of human BAT indicates that it is heterogeneous, composed of at least 3 types of functional adipocytes: “classical” brown, “inducible” brown (also known as “beige” [brown-like] or “brite” [“brown-in-white”]), and white adipocytes (17, 18). Molecular studies indicate that human supraclavicular fat consists of classical brown and beige/brite UCP1-positive adipocytes (19–22). Adrenergic activation of lipolysis stimulates the thermogenic activity of UCP1. Long chain fatty acids generated from the intracellular lipid pools are transported to the mitochondria through carnitine palmitoyltransferase 1 (CPT1) and used as a fuel source by brown adipocytes to produce heat (23–25). Additionally, free fatty acids (FFAs) have been proposed to act as allosteric activators of UCP1, releasing the purine nucleotide-mediated inhibition of UCP1 (24). In addition to fatty acids, circulating glucose can also be used by active BAT to fuel thermogenesis (26).

Among the 3 β-adrenergic receptor (β-AR) subtypes, β_3_-AR is the predominant receptor expressed in rodent brown adipocytes and is essential in regulating lipolysis and thermogenesis (27). Indeed, activation of β_3_-AR in rodents stimulates BAT glucose and FFA uptake (28, 29). There is controversy whether there is a physiological role of the human β_3_-AR in mediating these processes because of the relatively low levels of β_3_-AR mRNA when compared with rodent adipose tissue and poor cross-species selectivity of some β_3_-AR agonists (30–34). In contrast, β_1_-AR and β_2_-AR have both been proposed to regulate human lipolysis and thermogenesis (35, 36), with β_1_-AR increasing UCP1 expression in human multipotent-derived and immortalized brown adipocytes (37), while recently β_2_-AR has been proposed to be the main regulator of human thermogenesis (36).

However, several clinical studies have shown that adult human BAT and derived adipocytes express β_3_-AR mRNA (11, 20, 21), and activation of the β_3_-AR with the selective β_3_-AR agonist mirabegron (Myrbetriq) increases human BAT metabolic activity, whole-body energy expenditure, as well as plasma FFA and BAT glucose uptake (38–41). Obese subjects treated chronically with mirabegron show an increase in UCP1 expression in subcutaneous white adipose tissue (WAT) (41). Moreover, mutations in the gene encoding the β_3_-AR, *ADRB3*, have been correlated with insulin resistance, increased risk for obesity and diabetes, and nonalcoholic fatty liver disease in obese individuals (42–46).

Given the clinical evidence linking administration of the β_3_-AR agonist mirabegron and stimulation of BAT metabolic activity, we hypothesized that the β_3_-AR in human brown/beige adipocytes may play a role in thermogenesis and lipid metabolism. The present work evaluates the functional roles of β_3_-AR in differentiated primary adipocytes derived from human adipose tissue located in the supraclavicular fossa. We demonstrate that the β_3_-AR is necessary for maximum brown/beige adipocyte lipolysis and thermogenesis, emphasizing the importance of functional β_3_-ARs in the maintenance of brown/beige human adipocytes.

## Results

### Establishment of human primary supraclavicular adipocytes.

Adipose tissue from the superficial neck (SN) and supraclavicular (SCLV) regions was collected from the autopsy of a young woman. A higher expression of both UCP1 mRNA and protein was detected in the SCLV compared with SN fat from this subject (Supplemental Figure 1A; supplemental material available online with this article; https://doi.org/10.1172/jci.insight.139160DS1), along with markers for both brown and beige lineages, indicating that both thermogenic cell types were present in the SCLV fat (Supplemental Figure 1B) (19–22). Compared with SN, SCLV fat appeared to also have a higher expression of the β_1_-AR and β_3_-AR but similar levels of β_2_-AR (Supplemental Figure 1C). These data suggest that SCLV fat has bona fide brown adipocytes.

**Figure 1:**
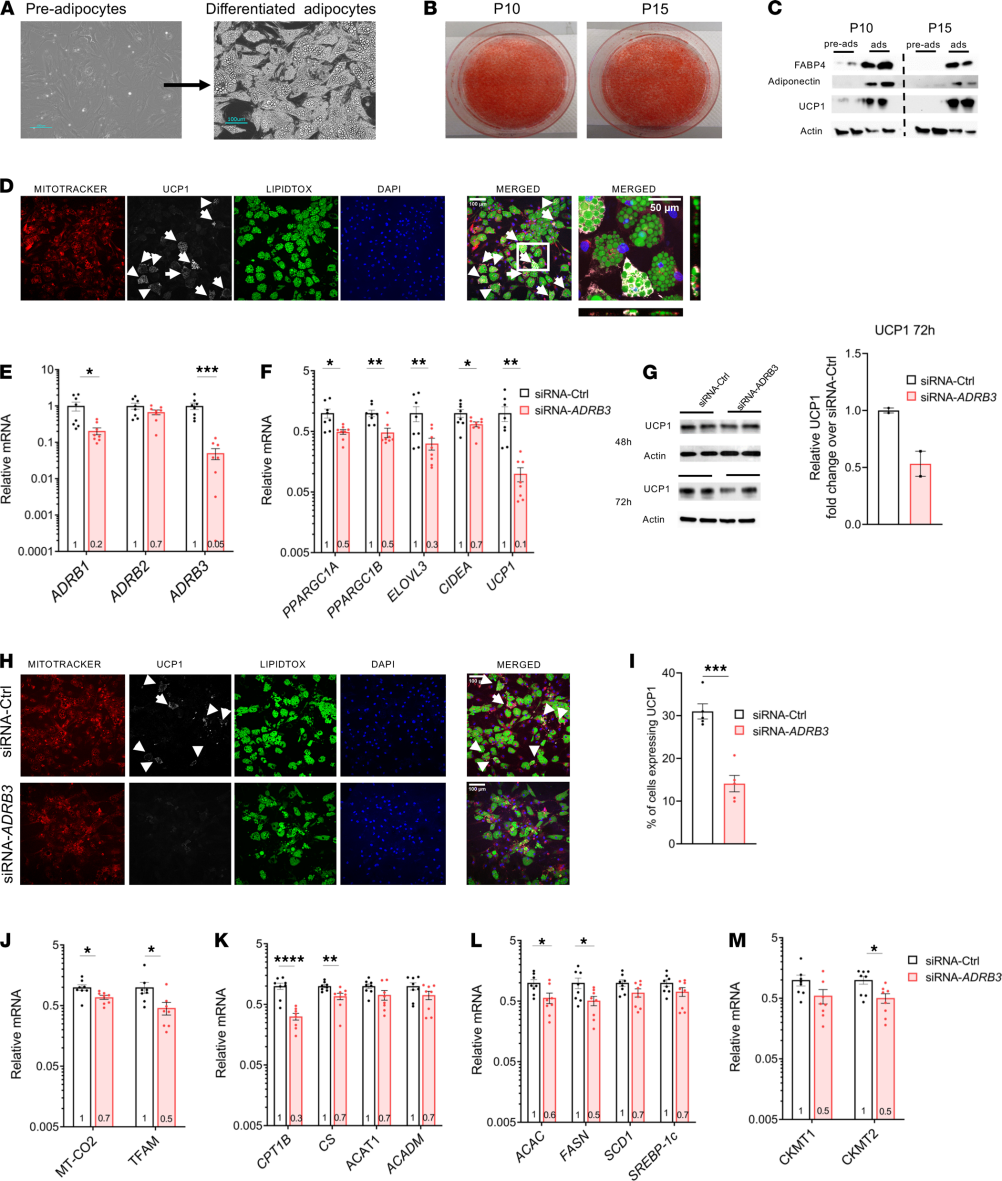
Establishment of primary human brown/beige adipocytes and the effects of silencing *ADRB3* on the cellular machinery. (**A**) Representative microscopic pictures of the morphology of undifferentiated and differentiated adipocytes. (**B**) Oil Red O staining of differentiated human primary adipocytes at passage number 10 (P10) and 15 (P15). (**C**) Protein expression of FABP4, adiponectin, and UCP1 at P10 and P15 in pre- and mature adipocytes. (**D**) Immunofluorescence of differentiated adipocytes staining for mitochondria (MitoTracker, red), lipid droplets (LipidTOX, green), anti-UCP1 (white), and nuclei (DAPI, blue). Scale bars: 100 μm for all images except the higher magnification, which is 50 μm. (**E** and **F**) The mRNA expression profiles of β-ARs (**E**) and thermogenic related genes (**F**) in transfected siRNA-control (siRNA-Ctrl) and siRNA-*ADRB3* adipocytes after 48 hours of transfection. (**G**) UCP1 protein levels in transfected adipocytes after 48 hours and 72 hours of transfection. (**H**) Immunofluorescence of siRNA-Ctrl and siRNA-*ADRB3* adipocytes stained with MitoTracker (red), LipidTOX (green), anti-UCP1 (white), and DAPI (blue). Scale bars: 100 μm. (**I**) Quantification of UCP1-positive adipocytes in siRNA-Ctrl and siRNA-*ADRB3* adipocytes from 5 sections. Arrows indicate cells expressing UCP1. (**J**–**M**) Expression levels of mtDNA-encoded gene (**J**), fatty acid oxidation genes (**K**), fatty acid synthesis genes (**L**), and creatinine kinase genes (**M**) in transfected siRNA-Ctrl and siRNA-*ADRB3* adipocytes. Gene expression data are normalized to siRNA-Ctrl adipocytes and expressed as mean ± SEM expression on a log_10_ scale. Data were analyzed by 2-tailed unpaired Student’s *t* test. ****P < 0.0001; ***P < 0.001; **P < 0.01; *P < 0.05.

Given the molecular characteristics of the SCLV fat, we isolated adipocyte progenitors and then cultured and differentiated them into mature adipocytes to determine the role of β_3_-AR in brown/beige cells. Upon exposure to adipogenic media, the primary preadipocytes transitioned from a fibroblast-like morphology to a mature adipocyte phenotype containing numerous lipid droplets (Figure 1, A and B, and Supplemental Figure 1D). Primary preadipocytes maintained their ability to duplicate and differentiate even at passage 15 (Figure 1, B and C). Therefore, all experiments were carried out between passages 8 and 15. To ensure that the polyclonal primary cells were brown/beige adipocytes, mRNA expression of peroxisome proliferator-activated receptor γ coactivator 1-α (*PPARGC1A*; PGC1α) and *UCP1* was analyzed over the differentiation progress of the cells. Compared with the preadipocyte stage, higher expression levels of both genes were detected starting at 5 days of induction treatment and were maintained over the course of differentiation (Supplemental Figure 1E). However, although *UCP1* gene expression was detected at day 5, UCP1 protein levels were detected starting at day 10 and were maintained throughout differentiation (Supplemental Figure 1F). This pattern is similar to what was observed in mouse brown adipocyte cell culture models (3, 47) where UCP1 gene expression precedes protein expression. Mitochondrial biogenesis during differentiation was monitored by examining the expression of the mitochondrial DNA–encoded (mtDNA-encoded) gene cytochrome c oxidase subunit 2 (*MT-CO2*) and the protein expression of the translocase of the outer membrane TOM20. Both components increased as preadipocytes differentiated into mature brown/beige adipocytes (Supplemental Figure 1, E and F). Moreover, compared with preadipocytes, transcription and translation levels of *FABP4*, *ADIPOQ*, and *CEBPA*, markers of later-phase differentiation, were substantially higher in mature, differentiated adipocytes (Figure 1C and Supplemental Figure 1G).

Along with *PPARGC1A* and *UCP1*, these adipocytes also exhibited higher levels of many brown/beige and thermogenic markers (Supplemental Figure 1H). mRNA levels for genes involved in fatty acid oxidation, namely *CPT1* and citrate synthase (*CS*), were also higher in mature adipocytes (Supplemental Figure 1I). The expression of all 3 β-ARs was detected in preadipocytes, and expression levels increased 58-, 27-, and 5-fold in mature adipocytes for *ADRB1*, *ADRB2*, and *ADRB3*, respectively (Supplemental Figure 1J). Staining with MitoTracker dye revealed an abundance of mitochondria in differentiated cells expressing UCP1, providing evidence for colocalization of UCP1 to the mitochondria (Figure 1D and Supplemental Figure 2A). In summary, the differentiated primary adipocytes from the SCLV region have the molecular machinery to support both lipolysis and thermogenesis. The adipocytes preserved the physiological properties that characterize their tissue of origin with high fidelity (48).

### Loss of β_3_-ARs in differentiated primary SCLV adipocytes alters the cellular thermogenic machinery.

Having established an in vitro model of human brown/beige adipocytes, we investigated the physiological role of the β_3_-AR in regulating lipolysis and thermogenesis by silencing *ADRB3* expression in differentiated adipocytes using lipofectamine-based transfection. Forty-eight hours after transfection of siRNA targeting *ADRB3* (siRNA-*ADRB3*), only 5% of *ADRB3* mRNA levels remained relative to control siRNA–transfected (siRNA-Ctrl–transfected) cells (Figure 1E). β_1_-AR mRNA levels were also lower, at 20% of control, while there was no change in β_2_-AR gene expression. The reduction in both β_3_-AR and β_1_-AR transcripts suggests either some degree of off-target binding by the siRNA-*ADRB3* or a physiological response by the cells as a consequence of reduced *ADRB3* mRNA or protein. To get a fuller picture of the effects of the specific silencing of *ADRB3*, we assessed the effects of targeting *ADRB1* with siRNA, which we report later in the manuscript.

Reduction of *ADRB3* expression led to substantial downregulation of genes involved in mitochondrial thermogenesis, including *PPARGC1A*, *PPARGC1B*, ELOVL fatty acid elongase 3 (*ELOVL3*), cell death inducing DFFA like effector a (*CIDEA*), and *UCP1*: 50%, 52%, 68%, 34%, and 90% reductions, respectively (Figure 1F). As observed during the differentiation of these adipocytes, lower *UCP1* mRNA levels at 48 hours preceded the reduction in UCP1 protein, which was more evident by 72 hours (Figure 1G). Absence of UCP1 is known to alter mitochondrial proteomics by reducing mitochondrial content and abundance of electron transport chain complexes inducing mitochondrial dysfunction (49). Thus, we assessed whether proteins of the oxidative complexes in the mitochondrial electron transport chain (ETC) and the mitochondria were affected by the lower *UCP1* expression in siRNA-*ADRB3* adipocytes. Expression of the nuclear encoded genes and proteins of each complex of the ETC were similar in both siRNA-*ADRB3* and control cells and independent of the absence or presence of UCP1 (Supplemental Figure 3, A and B). However, costaining with MitoTracker dye and an anti-UCP1 antibody revealed lower mitochondrial and UCP1 protein levels (Figure 1H and Supplemental Figure 2, B and C) in siRNA-*ADRB3* adipocytes. Compared with the siRNA-Ctrl adipocytes, the *ADRB3*-silenced adipocytes had a 55% decrease in UCP1-positive cells (Figure 1I) but similar number of cells that accumulated lipid droplets (Supplemental Figure 2D). At the same time, the expression of brown/beige adipocyte markers was similar in siRNA-*ADRB3* and in siRNA-Ctrl adipocytes, indicating no loss of their brown/beige phenotype (Supplemental Figure 3C).

Because brown/beige adipocytes utilize fatty acids to activate UCP1 and support thermogenesis, we measured the expression of key mitochondrial and fatty acid oxidation genes. In line with cells expressing less UCP1 protein in the siRNA-*ADRB3* brown/beige adipocytes, expression of mtDNA-encoded genes *MT-CO2* and transcription factor A, mitochondrial (*TFAM*), was also reduced, by 33% and by 54%, respectively (Figure 1J), suggesting lower mitochondrial quantity. However, among the genes and proteins of each ETC complex on the mitochondria, *COX7B*, which encodes for cytochrome c oxidase subunit 7B part of complex IV, was the only gene decreased (44%) in the *ADRB3*-transfected adipocytes (Supplemental Figure 3, B and C). Additionally, the expression of *CPT1*, the enzyme that catalyzes the initial reaction in the mitochondrial import of long chain fatty acids, was considerably lower (by 66%), as was the expression of *CS* (by 33%) (Figure 1K). The mRNA levels of acetyl-CoA acetyltransferase (*ACAT1*) and medium chain acyl-CoA dehydrogenase (*ACADM*), genes coding for enzymes that oxidize fatty acids, were not significantly affected (Figure 1K). Likewise, the expression of fatty acid synthesis genes acetyl-CoA carboxylase α (*ACAC*) and fatty acid synthase (*FASN*) were decreased (by 40% and 50%, respectively), while *SCD1* and *SREBP-1c* were not affected by *ADRB3* knockdown (Figure 1L). Phosphocreatine cycling has been shown to contribute to oxidative metabolism in human brown adipocytes (50). Therefore, we analyzed the transcript levels of mitochondrial creatine kinases *CKMT1* and *CKMT2* in the knockdown cells and observed a 50% decrease in both genes, but only the comparison with *CKMT2* yielded a *P* < 0.05 (Figure 1M).

Collectively, these data indicate that silencing of *ADRB3* in human brown/beige adipocytes alters the cellular thermogenic machinery and causes a reduction in the expression levels of genes associated with fatty acid metabolism, mitochondrial mass, and thermogenesis without compromising the brown/beige phenotype.

### Loss of β_3_-AR impairs SCLV adipocyte cAMP accumulation and β-AR–mediated lipolysis.

**Figure 2:**
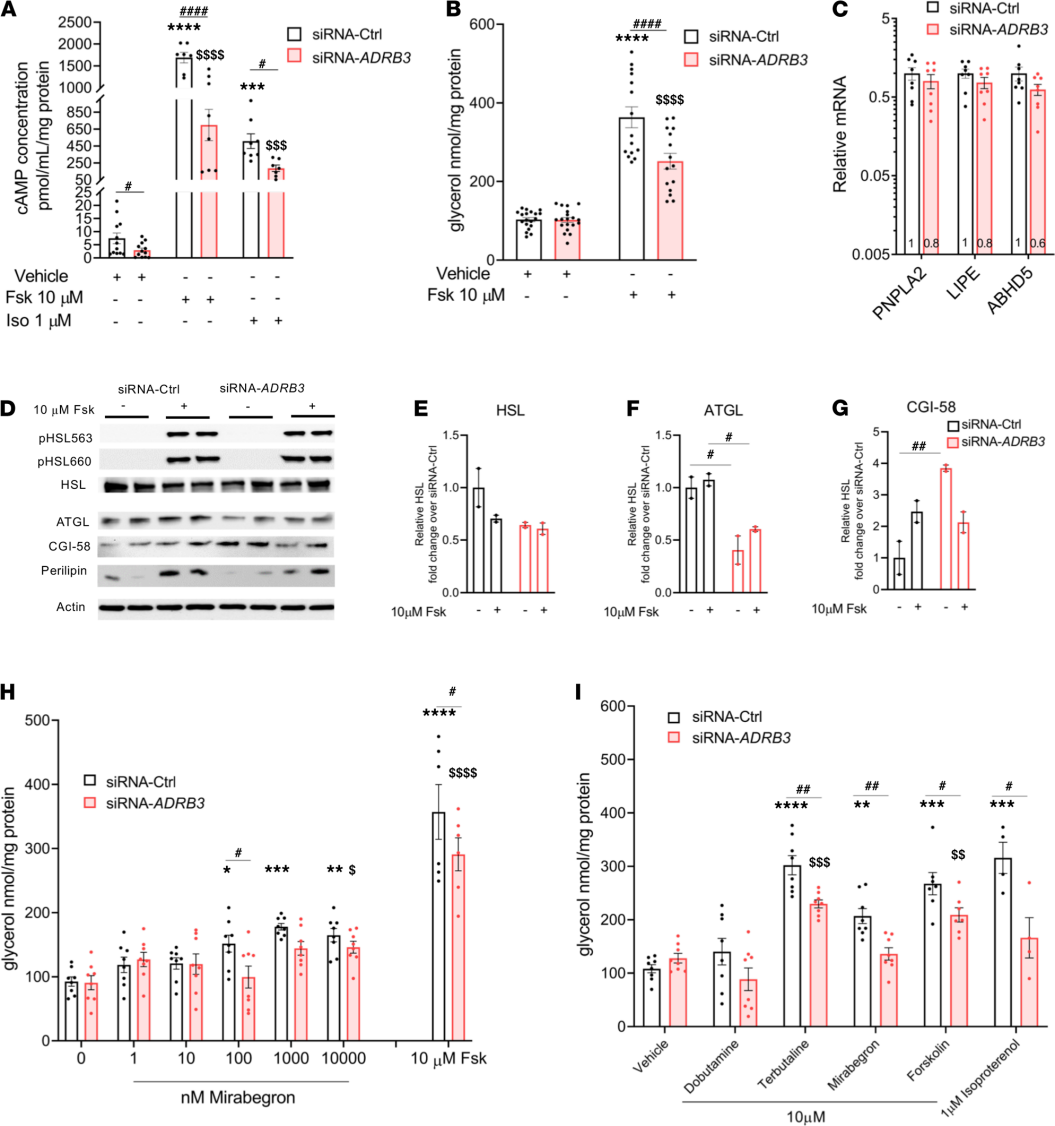
Silencing *ADRB3* in human brown/beige adipocytes lowers cAMP and affects β-AR–stimulated lipolysis. (**A** and **B**) Basal, forskolin-stimulated (Fsk 10 μM) and isoproterenol-stimulated (Iso 1 μM), cAMP concentration (**A**) and glycerol release (**B**) in siRNA-Ctrl and siRNA-*ADRB3* adipocytes. (**C**) RNA levels of lipolytic genes *PNPLA2*, *LIPE*, and *ABHD5* in siRNA-Ctrl and siRNA-*ADRB3* adipocytes. (**D**) Protein expression of pHSL563, pHSL660, HSL, ATGL, CGI-58, perilipin, and actin in cell lysates following Fsk treatment. (**E**–**G**) Quantification of HSL (**E**), ATGL (**F**), and CGI-58 (**G**) from Western blotting analysis. (**H**) Glycerol released into the incubation media following dose-dependent treatment with mirabegron (1-10-100-1000-10,000 nM) in siRNA-Ctrl– and siRNA-*ADRB3*–transfected adipocytes. (**I**) Glycerol released by siRNA-Ctrl and siRNA-*ADRB3* adipocytes treated with 10 μM of relatively selective human β_1_ agonist dobutamine, β_2_ agonist terbutaline, and β_3_ agonist mirabegron. Fsk (10 μM) and Iso (1 μM) were used as positive controls of agonist-induced lipolysis. Data are represented as mean ± SEM. Two-tailed unpaired Student’s *t* test and a 2-way ANOVA were used for statistical analysis. Gene expression data are normalized to siRNA-Ctrl adipocytes and expressed on a log_10_ scale. For cAMP and lipolysis data, *when comparing basal with stimulated in siRNA-Ctrl, ^$^when comparing basal with stimulated in siRNA-*ADRB3* adipocytes, ^#^when comparing same doses between siRNA-Ctrl and siRNA-*ADRB3* adipocytes. *^,$,#^*P* < 0.05; **^,$$,##^*P* < 0.01; ***^,$$$^*P* < 0.001; ****^,$$$$,####^*P* < 0.0001.

Having demonstrated the effects of silencing *ADRB3* on gene expression, we next assessed the functional implications. Since fatty acids are required for BAT thermogenesis (24, 51–54), we investigated the functional consequence of β_3_-AR deficiency on levels of the second messenger cAMP and lipolysis in *ADRB3*-silenced adipocytes. siRNA-*ADRB3* adipocytes had lower unstimulated cAMP levels (by 60%) compared with control cells. In the absence of ligand, suppression of *ADRB3* alone lowers adenylyl cyclase activity, possibly due to the disruption of precoupled stable complexes formed between the β-AR and adenylyl cyclase, which consequently lowers basal cAMP. Moreover, when treated with either forskolin (Fsk), a direct activator of AC, or isoproterenol (Iso), a pan–β-AR agonist, the adipocytes displayed a significantly lower ability to generate cAMP, by 40% and 55%, respectively, compared with the control cells (Figure 2A).

Next, we measured basal and Fsk-stimulated lipolysis and found no difference between the control and silenced *ADRB3* cells in unstimulated lipolysis. However, the siRNA-*ADRB3* adipocytes had reduced capacity to undergo lipolysis upon Fsk treatment, with a 70% decrease (Figure 2B), suggesting impairment in the downstream β-AR signaling. Therefore, we evaluated the gene expression of patatin like phospholipase domain containing 2 (*PNPLA2*); lipase E, hormone sensitive type (*LIPE*); and abhydrolase domain containing 5, lysophosphatidic acid acyltransferase (*ABHD5*), that respectively encode adipocyte triglyceride lipase (ATGL), hormone sensitive lipase (HSL), and CGI-58, a direct activator of ATGL. ATGL and HSL are the predominant lipases in BAT that catalyze the first 2 steps of triglyceride breakdown. There were no differences in the expression of these genes between the control and *ADRB3*-siRNA–transfected groups (Figure 2C). Immunoblotting showed similar levels of actin, perilipin, total HSL, and PKA-phosphorylated HSL (pHSL563 and pHSL660) in siRNA-*ADRB3* cells relative to siRNA-Ctrl adipocytes at baseline (Figure 2, D and E) and in response to Fsk (Figure 2D). In contrast, independent of Fsk treatment, ATGL protein levels were significantly lower in the siRNA-*ADRB3* adipocytes (Figure 2, D and F). However, the siRNA-*ADRB3* adipocytes had higher CGI-58 protein expression, which did not further increase upon Fsk treatment (Figure 2, D and G). This suggests a defect in the translation but not transcription of ATGL, CGI-58, and HSL enzymes. Lower levels of ATGL and higher levels of CGI-58 in the siRNA-*ADRB3* adipocytes could explain the reduced intracellular triglyceride lipolysis even after Fsk treatment, an activator of lipolysis acting downstream of the cell surface receptors.

β_1_-AR and β_2_-AR have been implicated in human WAT and BAT triglyceride hydrolysis (32, 35–37), while the involvement of the β_3_-AR remains controversial (55–61). To evaluate whether β_3_-ARs participate in the stimulation of lipolysis in human brown/beige adipocytes, we measured lipolysis in response to mirabegron, currently the only FDA-approved agonist selective for the human β_3_-AR. Glycerol released by the control and *ADRB3* cells was monitored following dose-dependent treatment with mirabegron. Compared with unstimulated lipolysis, in the siRNA-Ctrl adipocytes, mirabegron induced lipolysis at 100 nM, 1 μM, and 10 μM with 1.6-, 1.9- and 2.2-fold increases, respectively (Figure 2H). In contrast, in adipocytes with silenced *ADRB3*, mirabegron-induced lipolysis was increased only at 10 μM, and the increase was 1.7-fold higher than unstimulated lipolysis in siRNA-*ADRB3* adipocytes (Figure 2H). These data suggest that only at supraphysiological concentrations, mirabegron could be mobilizing FFA by binding to a combination of the small number of remaining β_3_-ARs in addition to the β_1_-ARs and β_2_-ARs.

We compared the effects of mirabegron on lipolysis with those caused by the relatively specific human β_1_-AR-and β_2_-AR agonists, dobutamine and terbutaline, and used Fsk and Iso as positive controls (61, 62). In primary brown/beige siRNA-Ctrl adipocytes, an increase in glycerol release in response to activation of the β_2_-AR with terbutaline (2.8-fold), β_3_-AR with mirabegron (1.9-fold), Fsk (2.5-fold), and Iso at 1 μM (2.3-fold) was detected (Figure 2I). Strikingly, these responses were blunted after treatment with siRNA-*ADRB3* (Figure 2I). These data indicate that intact *ADRB3* is required for normal signal transduction downstream to all 3 β-ARs and AC.

Besides reduced β_3_-AR signaling, the observed impairments in the thermogenic and lipolytic machinery could theoretically be attributed to the presence of an adrenergic receptor antagonist in the culture medium. To address this issue, we incubated nontransfected mature primary brown/beige adipocytes in the same transfection medium, without lipofectamine and siRNA, prior to treatment with propranolol, a β_1/2/3_-AR blocker. Propranolol did not have any effect on unstimulated lipolysis, showing that there was no adrenergic activator in the culture system (Supplemental Figure 4A). Furthermore, the increased glycerol release in response to all β-AR agonists was blunted in the presence of propranolol (Supplemental Figure 4A). Since siRNA-Ctrl and siRNA-*ADRB3* adipocytes are deprived of all hormones present within the differentiation cocktail when transfected, these data reinforce that the presence of *ADRB3* is regulating the lipolytic and thermogenic machinery as evident by the lower tonic levels of cAMP (Figure 2A) and thermogenic genes, including UCP1 (Figure 1, F and H).

### Silencing ADRB3 in human brown/beige adipocytes lowers lipolysis-activated UCP1-induced uncoupled and maximal respiration.

**Figure 3:**
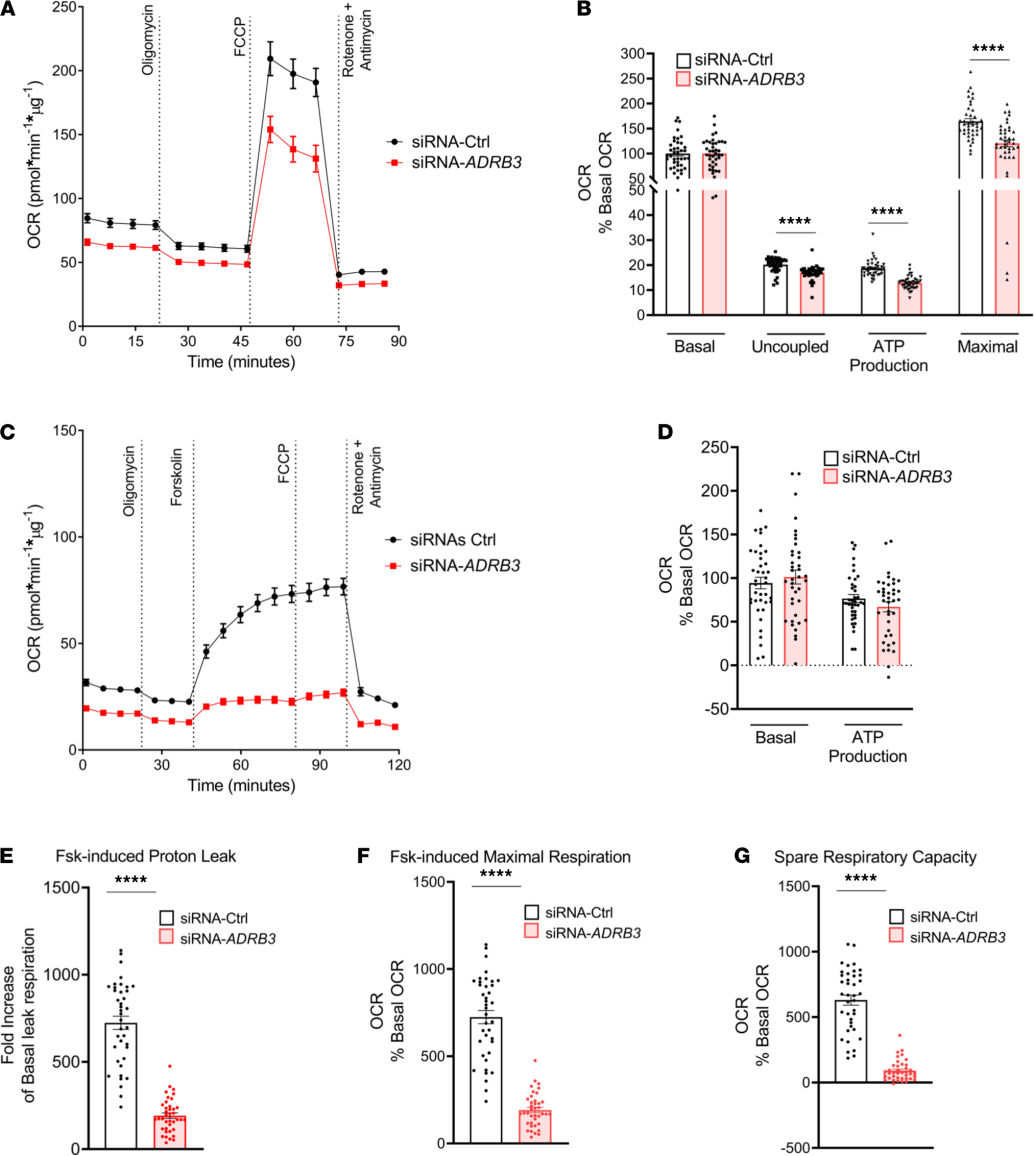
Silencing *ADRB3* in human brown/beige adipocytes lowers lipolysis-activated UCP1-induced uncoupled and maximal respiration. (**A** and **B**) OCR (**A**) and quantification of respiratory profile by differentiated siRNA-Ctrl and siRNA-*ADRB3* transfected adipocytes (**B**). (**C**–**G**) OCR trace (**C**), quantification of respiratory profile (**D**), Fsk-induced proton leak (**E**), and Fsk-induced maximal respiration (**F**) and spare respiratory capacity (**G**) in the presence of 2% BSA by differentiated siRNA-Ctrl– and siRNA-*ADRB3*–transfected adipocytes. Two-tailed unpaired Student’s *t* test was used for statistical analysis. ****P < 0.0001.

Humans treated with niacin, which inhibits WAT and BAT intracellular lipolysis, have severe thermoregulatory defects, indicating that lipolysis is necessary for BAT thermogenesis (63, 64). Therefore, we investigated cellular respiration in primary brown/beige adipocytes by subjecting the cells to a mitochondrial stress test. First, we monitored oxygen consumption rate (OCR) without the activation of lipolysis and found an overall lower OCR in the siRNA-*ADRB3*–transfected adipocytes (Figure 3A). Baseline respiration and nonmitochondrial respiration were, respectively, 8.4 pmol/min/μg and 9.4 pmol/min/μg lower in the siRNA-*ADRB3* adipocytes (Supplemental Figure 5A). We detected a significant impairment in the siRNA-*ADRB3* adipocytes to increase ATP-linked respiration by 5.7 pmol/min/μg (–3.4%), uncoupled respiration by 3.2 pmol/min/μg (–2%), and FCCP-induced maximal respiration by 44 pmol/min/μg (–27%) (Figure 3, A and B).

Next, to unambiguously examine the requirement of *ADRB3* in the lipolytic activity to activate UCP1 thermogenic activity, we used Fsk to stimulate lipolysis. UCP1, activated by fatty acids released from intracellular lipolysis, acutely increases the rate of proton leak produced by uncoupled respiration. We measured oligomycin-insensitive respiration and proton leak rate in transfected adipocytes under baseline and Fsk-stimulated conditions (Fsk-OLIGO-R+A) in the presence of bovine serum albumin (BSA), which prevents reesterification of extracellular fatty acids (65, 66). Nonmitochondrial respiration was 9.8 pmol/min/μg lower in the siRNA-*ADRB3* adipocytes (Supplemental Figure 5B). After normalizing for basal OCR (Figure 3, C and D), we found that treatment of the *ADRB3*-transfected cells with Fsk in the presence of 2% BSA had a lower proton leak by 13 pmol/min/μg (–58%) (Figure 3E), lower maximal respiration by 38 pmol/min/μg (–76%) (Figure 3F), and lower spare respiratory capacity by 38 pmol/min/μg (–87%) (Figure 3G). Silencing *ADRB3* in human beige/brown adipocytes significantly reduced Fsk-induced leak respiration, maximal respiration, and spare respiratory capacity, which confirms that functional *ADRB3* is required to fully increase uncoupled respiration. The lower spare respiratory capacity in the siRNA-*ADRB3* adipocytes also indicates mitochondrial dysfunction. Additionally, the lower nonmitochondrial respiration in the *ADRB3*-transfected adipocytes compared with their controls suggests that silencing of *ADRB3* could also affect nonmitochondrial sources of oxidation that are biologically relevant to maintain cellular respiration.

Together, these data indicate that functional *ADRB3* is required for lipolysis-induced fatty acid activation of UCP1-mediated uncoupled respiration in cultured brown/beige adipocytes.

### Silencing ADRB3 in human brown/beige adipocytes increase intracellular glycolytic flux for thermogenic activation.

**Figure 4:**
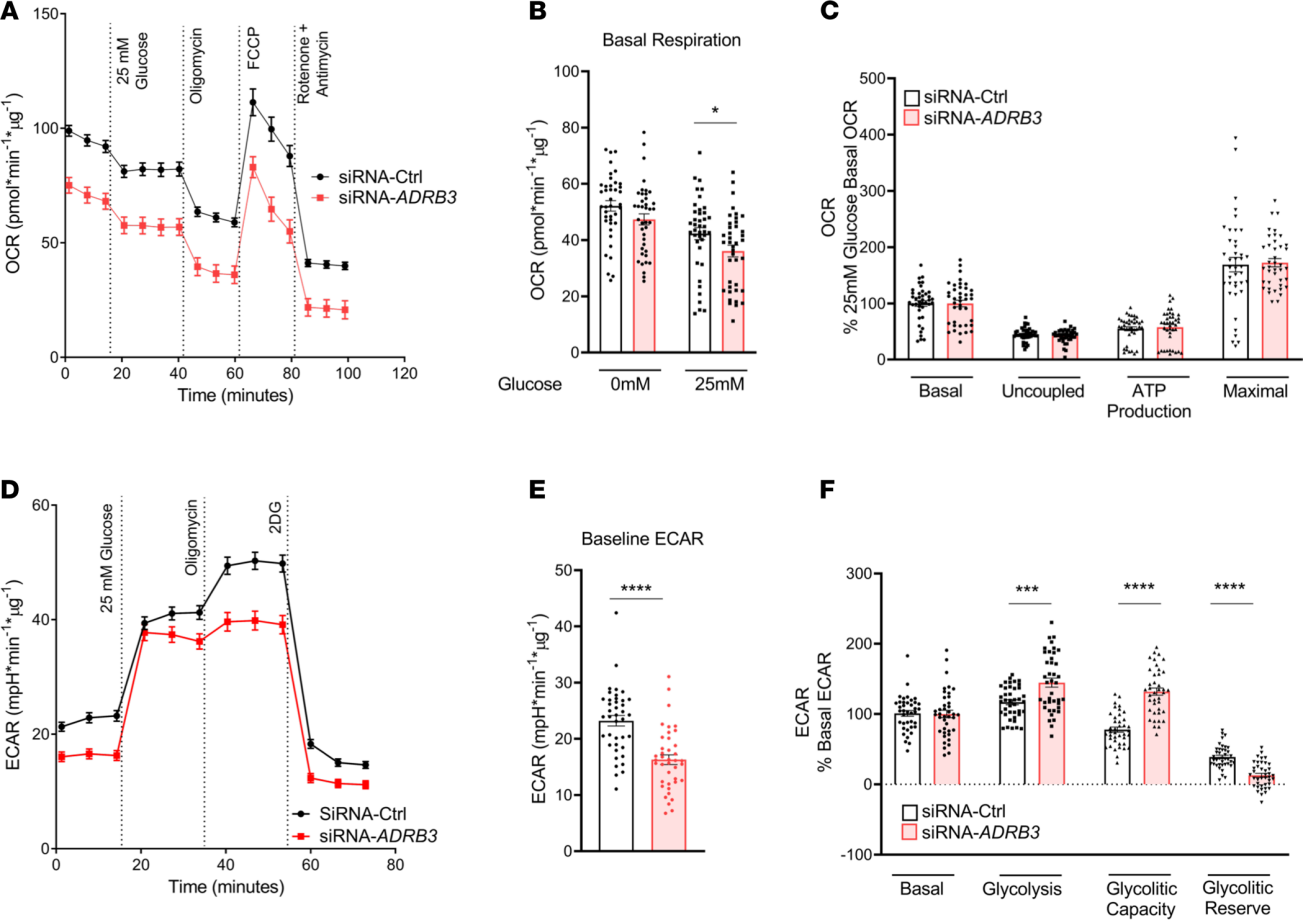
Silencing *ADRB3* in human brown/beige adipocytes increases intracellular glycolytic flux for thermogenic activation. OCR (**A**), quantification of basal respiration in the presence and absence of 25 mM glucose (**B**), and respiratory profile (**C**) in siRNA-Ctrl– and siRNA-*ADRB3*–transfected adipocytes when exposed to high glucose. (**D**–**F**) ECAR after sequential addition glucose (25 mM), oligomycin, and 2-deoxyglucose (**D**) and quantification of baseline ECAR (**E**) and glycolysis and glycolytic capacity and reserve (**F**) in siRNA-Ctrl and siRNA-*ADRB3* adipocytes. Data are represented as mean ± SEM. Two-tailed unpaired Student’s *t* test and a 2-way ANOVA were used for statistical analysis. *P < 0.05; ***P < 0.001; ****P < 0.0001.

Glucose is used as a thermogenic and lipogenic substrate by BAT (28, 67), so we assessed the requirement of brown/beige adipocytes’ glucose uptake and subsequent glycolysis using OCR and extracellular acidification rate (ECAR) in response to an acute and high concentration of glucose (25 mM). In response to glucose, the siRNA-*ADRB3* adipocytes had a lower basal OCR by 6 pmol/min/μg (by 15%) compared with the siRNA-Ctrl cells (Figure 4, A and B). However, when converted with the basal glucose OCR of each transfection treatment, the siRNA-*ADRB3* adipocytes displayed a similar ability to increase proton leak, ATP production, and FCCP-induced maximal glucose substrate oxidation compared with the control transfected adipocytes (Figure 4C). Expression of the principal cell surface glucose transporters GLUT1 and GLUT4 for glucose uptake, respectively encoded by *SLC2A1* and *SLC2A4*, was not different in the siRNA-*ADRB3* and siRNA-Ctrl adipocytes (Supplemental Figure 5C).

Next, we monitored glucose-induced glycolysis, the metabolic pathway that converts glucose into pyruvate or lactate, to determine whether glucose is properly metabolized for mitochondrial ATP production. The nonglycolytic acidification rate, which is reflected by the ECAR in the absence of glucose, was lower in the siRNA-*ADRB3* adipocytes compared with the siRNA-Ctrl cells by 6 mpH/min/μg (–30%) (Figure 4, D and E). Glycolysis, measured after the injection of glucose, was adjusted for the nonglycolytic acidification rate to represent the ECAR from glycolysis. After adjustment, compared with the siRNA-Ctrl adipocytes, the siRNA-*ADRB3* adipocytes had higher glycolysis (+28%). They also had higher glycolytic capacity, defined as the cell’s ability to sustain ATP demand solely by glycolysis, which is achieved by the injection of oligomycin that blocks ATP synthase (+54%) (Figure 4F). In addition, there was lower glycolytic reserve (–26%), indicating a lower ability of the siRNA-*ADRB3* adipocytes to convert glucose into pyruvate or lactate in response to an acute increase in energy demand. Overall, this suggests that the siRNA-*ADRB3* adipocytes switch to glycolysis when energy demand increases to augment glucose consumption for mitochondrial ATP production and respiration to achieve thermogenic activation.

### Silencing ADRB1 in primary brown/beige adipocytes decreases UCP1 expression but does not decrease lipolysis or cellular respiration.

**Figure 5:**
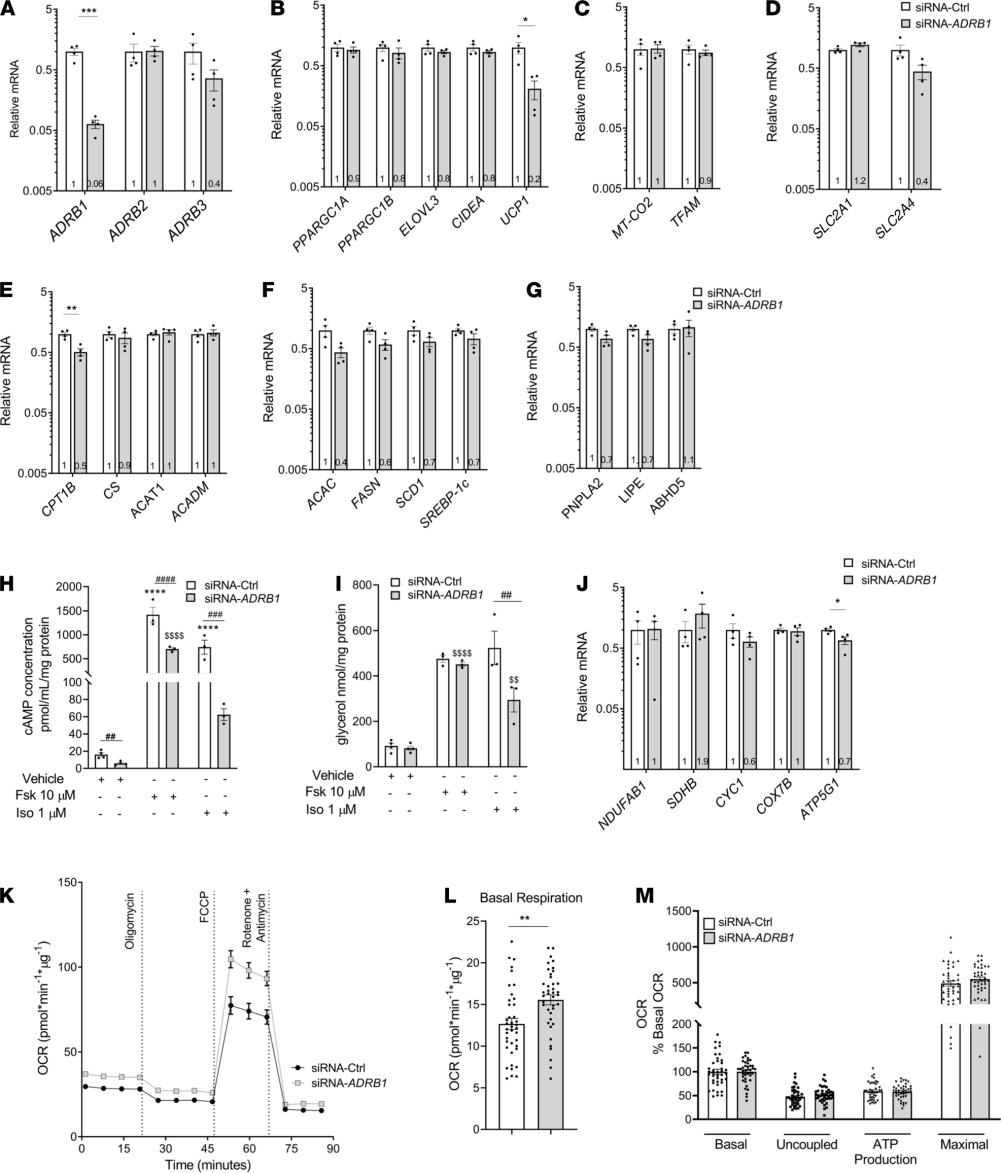
Silencing *ADRB1* in primary brown/beige adipocytes decreases UCP1 expression but does not decrease lipolysis or cellular respiration. (**A**–**G**) The mRNA expression profiles of β-ARs (**A**), thermogenic related genes (**B**), mtDNA-encoded genes (**C**), glucose transporters (**D**), fatty acid oxidation genes (**E**), fatty acid synthesis genes (**F**), and lipolytic genes *PNPLA2*, *LIPE*, and *ABHD5* (**G**) in siRNA-Ctrl and siRNA-*ADRB1* adipocytes after 48 hours of transfection. (**H** and **I**) Fsk-stimulated (10 μM) and Iso-stimulated (1 μM) cAMP (**H**) and glycerol release (**I**) in siRNA-Ctrl and siRNA-*ADRB1* adipocytes. (**J**) RNA levels of nuclear encoded ETC genes in siRNA-Ctrl and siRNA-*ADRB1* adipocytes. (**K**–**M**) OCR trace (**K**) and quantification of basal respiration (**L**) and respiratory profile (**M**) by differentiated siRNA-Ctrl– and siRNA-*ADRB1*–transfected adipocytes. Data are represented as mean ± SEM. Two-tailed unpaired Student’s *t* test and a 2-way ANOVA were used for statistical analysis. Gene expression data are normalized to siRNA-Ctrl adipocytes and expressed on a log_10_ scale. For cAMP and lipolysis data *when comparing basal with stimulated in siRNA-Ctrl, ^$^when comparing basal with stimulated in siRNA-*ADRB1* adipocytes, ^#^when comparing same doses between siRNA-Ctrl and siRNA-*ADRB1* adipocytes. *P < 0.05; **^,$$,##^*P* < 0.01; ***^,###^*P* < 0.001; ****^,$$$$,####^*P* < 0.0001.

Having observed that silencing *ADRB3* also affects *ADRB1* expression (Figure 1E), we selectively silenced *ADRB1* (siRNA-*ADRB1*) in order to dissect how much of the cellular and molecular effects were contributed by a near-total absence of *ADRB1* expression in primary human brown/beige adipocytes. Gene expression data of the β-ARs indicated a successful knockdown of *ADRB1* (94%) without significant reductions in *ADRB2* or *ADRB3* (64%) mRNA levels (Figure 5A). Except for *UCP1*, where a 79% decrease was detected in the siRNA-*ADRB1* adipocytes, no differences were found in the other thermogenic genes analyzed (Figure 5B), in the mtDNA-encoded genes (Figure 5C), or in the glucose transporters (Figure 5D). Among the fatty acid oxidation and synthesis genes analyzed, only the expression of *CPT1* was lowered in the siRNA-*ADRB1* adipocytes (by 49%) (Figure 5, E and F). Considering the different effects of silencing RNA to reduce expression of *ADRB3* and *ADRB1*, these data indicate that the presence of *ADRB3* in human brown/beige adipocytes is necessary for the full expression of genes essential for the cellular thermogenic machinery, fatty acid metabolism, mitochondrial mass, and thermogenesis.

In the siRNA-*ADRB3* adipocytes, basal and Fsk- and Iso-stimulated cAMP secretion was diminished compared with their siRNA-Ctrl adipocytes. Silencing *ADRB1* in primary human brown/beige adipocytes also lowered unstimulated and Fsk- and Iso-stimulated cAMP production by 64%, 51%, and 92%, respectively (Figure 5H). However, while unstimulated and Fsk-induced lipolysis were unaffected, the Iso-induced lipolysis was decreased by 44% (Figure 5I). This suggests that *ADRB1* may not be the primary receptor regulating the lipolytic machinery and mediating lipolysis in human brown/beige adipocytes. Similar to the siRNA-*ADRB3* adipocytes, no differences were detected in the expression of *PNPLA2*, *LIPE*, and *ABHD5* (Figure 5G). Together, these data further convey the importance of *ADRB3* in primary brown/beige adipocytes in maintaining functional intracellular lipolytic machinery. To further dissect the contribution of β_1_-AR and β_3_-AR on lipolysis and determine the functional profile of loss of the β_3_-AR, we used L-748,337 (68, 69), one of the very few human β_3_-AR antagonists available. Pretreatment with L-748,337 followed by treatment with β-AR agonists had no effects on basal lipolysis, but L-748,337 significantly reduced lipolysis when adding β_1_-, β_2_-, or β_3_-AR agonists (Supplemental Figure 4A). These data are therefore consistent with the reduction in lipolysis seen with the siRNA-*ADRB3* adipocytes.

Next, we established the effects of silencing *ADRB1* adipocytes on OCR in siRNA-*ADRB1* adipocytes and observed a significantly higher respiratory rate in the siRNA-*ADRB1* adipocytes compared with control cells (Figure 5K). Basal respiration in the siRNA-*ADRB1* adipocytes was 23% higher compared with the control cells (Figure 5L). When adjusted to basal OCR, siRNA-*ADRB1* and control transfected adipocytes displayed similar ability to perform uncoupled respiration, produce ATP, and increase FCCP-stimulated and maximal respiration (Figure 5M). Analysis of the ETC complex genes showed a decrease in the expression of *ATP5G1*, which encodes membrane subunit c of the mitochondrial ATP synthase (33%) (Figure 5J). The increase in cellular respiration and loss of *ATP5G1* expression in the siRNA-*ADRB1* could be allowing protons to leak into the matrix or increase electron slippage that could result in increased OCR in the absence of proton translocation.

Given that silencing *ADRB3* lowered OCR, but silencing *ADRB1* increased OCR, these data provide additional support that upon siRNA-A*DRB3* treatment, it is the reduction in the β_3_-AR that leads to the decreased thermogenic capacity in human brown/beige adipocytes.

### Silencing ADRB3 in immortalized human brown/beige adipocytes compromises the cellular lipogenic and thermogenic machinery.

Although primary adipocytes model the physiological behavior occurring in vivo, there is a limited supply. Therefore, we investigated whether silencing *ADRB3* in immortalized human brown preadipocytes from a separate subject (70) would affect UCP1 expression, lipolysis, and the thermogenic machinery. Similar to the primary adipocytes, and as previously reported (71), upon differentiation the immortalized brown adipocytes increased lipid accumulation (Supplemental Figure 6, A and B) and expression of late markers of differentiation (Supplemental Figure 6, C and E). The immortalized brown adipocytes displayed higher expression of brown adipocyte, thermogenic, mitochondrial markers (Supplemental Figure 6, D–F) and β_1_-AR and β_3_-AR levels (Supplemental Figure 6G).

Given that primary and immortalized undifferentiated and mature brown/beige adipocytes expressed comparable mRNA of the β-ARs and *UCP1* (Supplemental Figure 6H), we transfected the immortalized adipocytes with siRNA-*ADRB3* and observed similar reduction of mRNA levels of *ADRB3* (by 83%) (Supplemental Figure 7A); all thermogenic markers analyzed, *PPARGC1A*, *PPARGC1B*, *ELOVL3*, *CIDEA*, and *UCP1* by 48%, 77%, 83%, 79%, and 90% (Supplemental Figure 7B); and fatty acid metabolism genes *CS* (by 31%) and *SCD1* (by 51%) (Supplemental Figure 7, C and D). Unlike the primary brown/beige adipocytes, no changes were detected in the mtDNA-encoded genes (Supplemental Figure 7E), while significant decreases in the gene expression of *SLC2A4* (by 78%), *PNPLA2* (by 67%), and *LIPE* (by 53%) were detected in the siRNA-*ADRB3* immortalized brown adipocytes (Supplemental Figure 7, F and G). Levels of cAMP (Supplemental Figure 7H) in the *ADRB3*-silenced, immortalized adipocytes was lower when treated with Fsk by 24% compared with their controls. In conjunction with lower stimulated Fsk cAMP levels, the siRNA-*ADRB3*–transfected cells also had lower levels of p-PKA substrates (Supplemental Figure 7I) compared with their controls. Moreover, Fsk-stimulated lipolysis was also lower by 42% in the transfected cells, which was mainly due to impaired PKA-mediated activation of its HSL (pHSL563 and pHSL660), while no difference was detected in ATGL expression as in the primary cells (Supplemental Figure 7, J and K).

Cellular bioenergetics measured following mitochondrial metabolic stress showed that siRNA-*ADRB3*–treated immortalized brown adipocytes had lower basal respiration by 19% and 22% compared with the control and siRNA-*ADRB1* adipocytes, respectively. In contrast, the siRNA-*ADRB1* adipocytes displayed higher basal respiration, by 3% and 22%, compared with the corresponding siRNA-Ctrl and siRNA-*ADRB3* adipocytes (Supplemental Figure 7, L and M). When corrected for basal cellular respiration, siRNA-*ADRB3* immortalized adipocytes had a lower proton leak (–10%), a lower ATP production (–6%), and a lower maximal respiration (–29%) compared with the control adipocytes. On the other hand, the siRNA-*ADRB1* adipocytes had a proton leak and ATP production similar to the siRNA-Ctrl cells but an increased FCCP-induced maximal respiration by 50% (Supplemental Figure 7, L–N). When comparing the silenced *ADRB1* and *ADRB3* adipocytes, we observed a lower proton leak (–30%), a higher ATP production (+19%), and a higher maximal respiration (+39%) in the siRNA-*ADRB1* adipocytes (Supplemental Figure 7, L–N). These data highlight that with either in vitro model system, the β_3_-AR plays an extensive role in human brown adipocyte lipolysis and thermogenesis.

### Mirabegron uncouples respiration in human brown/beige adipocytes, and ADRB3 is required for functional ADRB1 and ADRB2 signaling.

To corroborate that a reduction of functional *ADRB3* affects respiratory capacity in human brown/beige adipocytes, we silenced *ADRB3*, treated the cells with mirabegron, and monitored proton dynamics.

**Figure 6:**
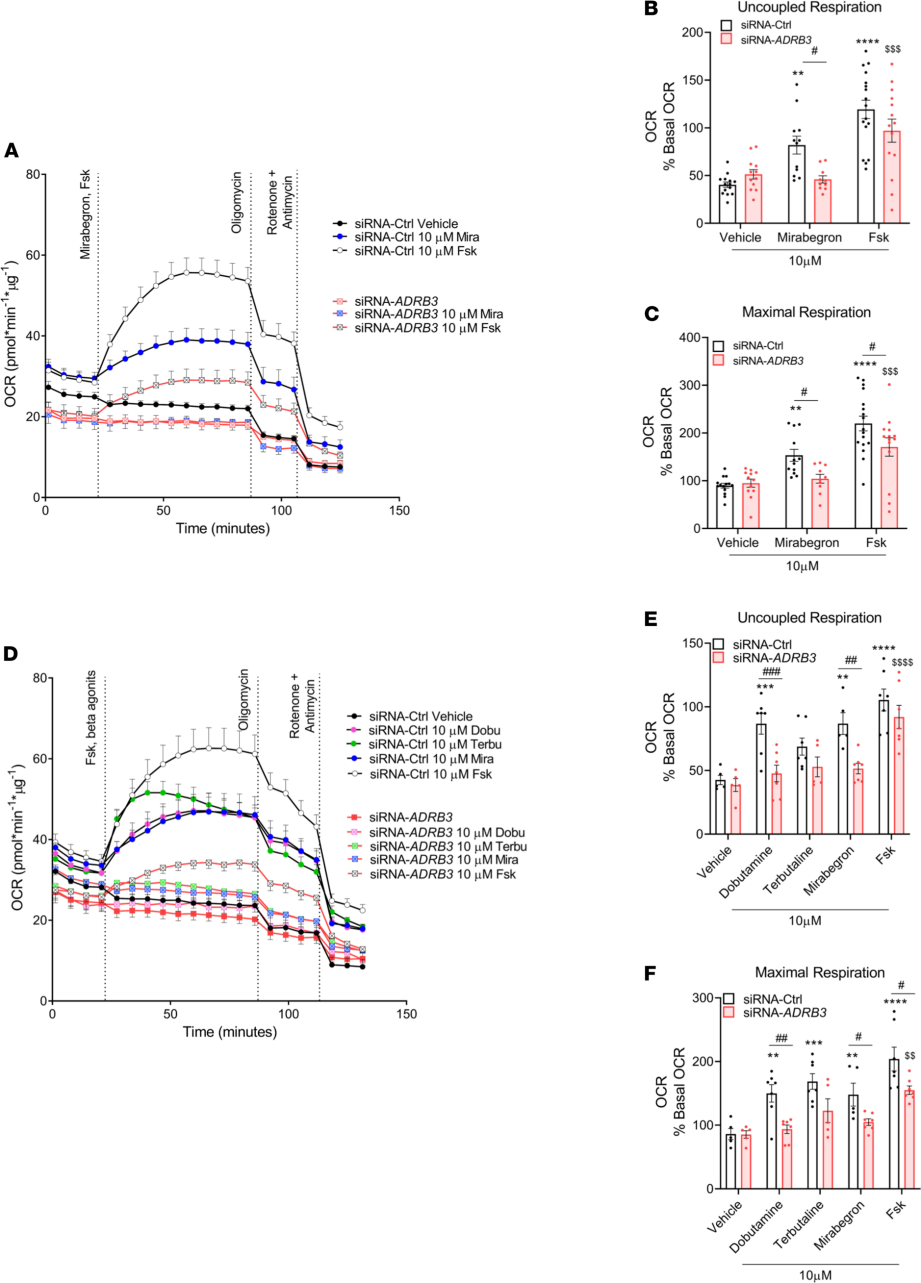
Mirabegron uncouples respiration in primary human brown/beige adipocytes, and *ADRB3* is required for functional *ADRB1, ADRB2* and downstream β-adrenergic signaling. (**A**–**C**) OCR trace (**A**) and quantification of uncoupled (**B**) and maximal respiration (**C**) in siRNA-Ctrl– and siRNA-*ADRB3*–transfected adipocytes in the presence of 2% BSA when stimulated with 10 μM mirabegron and Fsk. (**D**) Time course of OCR of control transfected and siRNA-*ADRB3* cells after 10 μM dobutamine, terbutaline, mirabegron, and Fsk treatment. (**E** and **F**) Quantification of uncoupled (**E**) and maximal respiration (**F**) in the presence of 2% BSA. All data presented are mean values ± SEM. Two-way ANOVA was used for statistical analysis: *when comparing basal to stimulated in siRNA-Ctrl, ^$^when comparing basal to stimulated in siRNA-*ADRB3* adipocytes, ^#^when comparing same doses between siRNA-Ctrl and siRNA-*ADRB3* adipocytes. ^#^*P* < 0.05; **^,$$,##^*P* < 0.01; ***^,$$$,###^*P* < 0.001; ****^,$$$$^*P* < 0.0001.

To verify whether mirabegron can increase the rate of proton leak acting through *ADRB3*, we measured oligomycin-insensitive respiration and proton leak rate in transfected adipocytes under basal and mirabegron- or Fsk-stimulated conditions (Mira/Fsk-OLIGO-R+A) in the presence of BSA. Control siRNA adipocytes had higher levels of basal respiration compared with the *ADRB3*-silenced adipocytes (Figure 6, A–C). In response to mirabegron, the siRNA-Ctrl adipocytes augmented their uncoupled respiration (Figure 6B) and maximal respiration (Figure 6C) by 102% and by 70%, respectively, while Fsk augmented both parameters by 196% and 145%, respectively, compared with the siRNA-Ctrl vehicle treatment. In contrast, mirabegron failed to increase either parameter in siRNA-*ADRB3* adipocytes (Figure 6, A–C). In fact, in *ADRB3*-transfected cells, mirabegron produced a similar response to their respective vehicle-treated cells, while Fsk augmented proton leak only by 89% and maximal respiration by 80% compared with the siRNA-*ADRB3* vehicle-treated cells. These data indicate that mirabegron, acting via the β_3_-AR, promotes thermogenesis in primary human brown/beige adipocytes.

To determine the contributions of the 3 β-ARs in thermogenesis, we treated siRNA-Ctrl adipocytes with β_1_-AR and β_2_-AR agonists that induce lipolysis, and we observed substantial increases in their cellular respiration (Figure 6D). Specifically, in the siRNA-Ctrl adipocytes, dobutamine and terbutaline each increased uncoupled respiration, respectively, by 104% and by 61% (Figure 6E) and maximal respiration (Figure 6F) by 74% and by 96%. However, the siRNA-*ADRB3–*treated brown/beige adipocytes did not respond to either β_1_-AR or β_2_-AR agonists to increase their cellular respiration (Figure 6, D–F). These data emphasize that (a) the activation of all 3 β-ARs is involved in prompting thermogenesis in human brown/beige adipocyte and that (b) *ADRB3* plays a crucial role in preserving an intact and functional downstream β-AR signaling cascade.

## Discussion

Given the relative size and accessibility of iBAT in rodents, most of the insights into brown adipocyte cellular physiology come from studying this depot. In mice, iBAT β_3_-ARs are the principal mediators of lipolysis and thermogenesis (1–3). A role for the β_3_-ARs in human energy metabolism is supported by clinical trials reporting associations between specific polymorphisms in the human *ADRB3* gene and higher rates of obesity, insulin resistance, and diabetes (42–44). Nevertheless, controversy remains with the functional relevance of the human β_3_-ARs (72, 73). Clarifying the picture has been hampered by the difficulty in accessing human BAT, the lower BAT β_3_-AR receptor levels compared with mice, and differences in murine versus human β_3_-AR ligand affinities. In the current study, we addressed several of these challenges by using human primary brown/beige adipocytes that express the β_3_-AR and by treating them with an FDA-approved, human-selective β_3_-AR agonist, mirabegron. Indeed, the polyclonal primary cells used in this study, isolated from a young female, exhibited high proliferative and adipogenic capacity. Like differentiated human multipotent adipocyte-derived stem cells (74), differentiated adipocytes expressed brown/beige adipocyte markers along with β_1_-, β_2_-, and β_3_-ARs. Moreover, expression levels of UCP1 mRNA and protein were much higher in differentiated adipocytes, closely recapitulating the profile of human BAT. These primary brown/beige adipocytes were functional, responding to adrenergic stimulation to induce lipolysis and thermogenesis.

To define the role of the β_3_-AR in human brown/beige adipocyte metabolism, we silenced *ADRB3* expression and saw an overall decrease in expression of genes relevant to thermogenesis and fatty acid metabolism. These changes were associated with lower production of cAMP and lipolysis even in the setting of AC activation with Fsk or various β agonists. The effects of the β_3_-AR silencing extended to decreased basal-, FCCP-, and lipolysis-induced cellular respiration. Moreover, although glucose uptake was maintained, glycolysis increased in the siRNA-*ADRB3* adipocytes, likely to compensate for the inability of the cell to undergo uncoupled respiration during thermogenesis. A decrease in *ADRB1* was also detected in these *ADRB3*-silenced adipocytes. To examine the individual contributions of *ADRB3* and *ADRB1* silencing to these effects, we silenced the *ADRB1* gene in differentiated primary brown/beige adipocytes. We found lower basal and stimulated cAMP, but no effects on Fsk-induced lipolysis, only on Iso-induced lipolysis. Cellular respiration in these cells was higher, rather than lower, as observed with the siRNA-*ADRB3* adipocytes. The impaired lipolysis and thermogenesis observed in the siRNA-*ADRB3* adipocytes were likely due to changes only in β_3_-AR function. These observations were recapitulated in an independently derived, immortalized human brown adipocyte cell line from a separate subject. Therefore, our findings in human adipocytes generated from the SCLV fat indicate that β_3_-ARs are necessary for normal human brown/beige lipolysis and thermogenesis and that β_3_-ARs are functionally involved in cellular energy metabolism.

In our primary cell model, at the concentration of agonists used, activation of β_2_- and β_3_-ARs significantly increased lipolysis, while the reduction in the expression of *ADRB3* in these cells compromised the lipolytic response, emphasizing a role for *ADRB3* in retaining a functional lipolytic machinery. Adenosine, which is produced by human brown adipocytes and can itself activate brown adipocytes by exerting inhibitory effects on AC (75), was not likely implicated in the effects on the lipolytic response as the brown/beige adipocytes were treated with adenosine deaminase and an adenosine receptor agonist (phenyl isopropyl adenosine, PIA). These measures were employed to rule out the contribution of extracellular adenosine acting on adenosine receptors, further strengthening the relationship between activation of the β-ARs and the lipolytic response in human brown adipocytes.

Blocking of β_1_-and β_2_-ARs by administration of propranolol reduced BAT activity measured by FDG-PET imaging (76, 77). Activation of β_3_-ARs increased BAT activity measured by the same approach (38–41). These clinical studies suggest that all 3 β-ARs can activate human thermogenesis (35–41). However, unlike the human β_3_-AR, the expression of which is restricted mostly to the urinary bladder, gallbladder, and BAT (39), the β_1_-AR is highly expressed throughout the cardiovascular system, and the β_2_-AR is found in the pulmonary airways, throughout the vasculature, and in skeletal muscle (78, 79). Therefore, it is difficult to use either β_1_- or β_2_-AR agonists to study human BAT physiology without causing prohibitive adverse events. When uncoupled respiration was tested using all 3 agonists on primary human brown/beige adipocytes, we found that β_1_- and β_2_-AR agonists produced the highest increase in cellular respiration, followed by the β_3_-AR, affirming that indeed all 3 receptors participate in prompting BAT thermogenesis. However, β_1_-AR and β_2_-AR undergo desensitization and are internalized, limiting their utility for chronic treatment (78). Of note, silencing the *ADRB3* inhibited the thermogenic effects triggered not just by mirabegron, a β_3_-AR agonist, but also by the other 2 β-AR agonists, indicating that the presence of functional β_3_-ARs is required for conserving a functional lipolytic and thermogenic machinery in brown/beige adipocytes. Additionally, these data suggest that these commonly used β_1_- and β_2_-AR agonists, dobutamine and terbutaline, could be partially activating and acting through *ADRB3*. Indeed, these agonists have been shown to have binding affinity for and activity at the β_3_-ARs (80, 81).

Our data emphasize that mirabegron (82), a drug in current clinical use, increases lipolysis and thermogenesis in human brown/beige adipocytes specifically through activation of the β_3_-AR. These data are in accordance with our clinical studies where we observed that acute mirabegron treatment increases FFA and energy expenditure (38, 39). In considering the mechanism by which even unstimulated β_3_-ARs, with no bound ligand, activate AC to produce cAMP and raise *UCP1* expression, we hypothesize that this phenomenon could occur because of protein/protein interactions or protein stability control. We postulate that the changes seen in basal metabolic processes in the *ADRB3*-silenced adipocytes were likely caused by a loss of low-level, tonic activation of the β_3_-AR and its precoupling with adenylyl cyclase (83–85), which is supported by the marked lower levels of basal and stimulated cAMP accumulation and ligand-stimulated lipolysis. Consequently, silencing the *ADRB3* is sufficient to lower the brown adipocyte’s ability to properly undergo lipolysis and thermogenesis. Future studies should determine the specific signaling pathways by which the β_3_-AR supports the core lipolytic and thermogenic roles of brown adipocytes. For example, it is conceivable, as previously hypothesized (86), that activation of the β_3_-AR in brown adipocytes could generate a signaling molecule, other than fatty acids, that directly activates UCP1 expression and function. Although the mechanistic link between mutations/polymorphisms in *ADRB3* and metabolic disease in humans remains to be established (87), it will be important to determine whether administration of a β_3_-AR agonist could be used to treat these conditions.

Even as we provide new insights into the role of β_3_-ARs in human brown/beige adipocyte function, this study has certain limitations. The in vitro experiments were conducted using polyclonal primary human preadipocytes that generate upon differentiation a mixture of white, beige/brite, and brown adipocytes. As such, we chose to study adipocytes derived from the SCLV and deep neck fat, which contains the highest number of UCP1-positive cells (71, 88). While our evaluation indicated that these cells have great utility in studying human BAT, the preadipocytes may differ from BAT cells in vivo and can even vary internally across serial passages. Additionally, even though the SMARTpool technology that combines 4 gene-specific siRNAs targeting *ADRB3* to minimize off-target effects, we detected a decrease in *ADRB1*, suggesting either some degree of off-target binding by the siRNA-*ADRB3* or a physiological response by the cells as a consequence of reduced *ADRB3* mRNA or protein. Moreover, the siRNA approach achieved more than 90% knockdown of β_3_-AR RNA, as opposed to other methods that can achieve near-total elimination of the receptor. On a related point, the lack of selective β_3_-AR antibodies precludes determination of the cell surface expression of each β-AR, making it more challenging to determine the absolute and relative contributions of the β_3_-AR to cellular physiology. Last, despite investigating primary adipocytes, these were derived from a single subject, so we repeated our key experiments on immortalized human brown preadipocytes from a separate subject to validate our observations.

In summary, our findings demonstrate that the β_3_-AR is present in human BAT depots and that primary preadipocytes differentiate into functional brown/beige cells. The β_3_-AR in human brown/beige adipocytes is important not just for initiating adrenergic signaling — its very presence plays a pivotal role in proper maintenance of downstream lipolytic and thermogenic machinery. Finally, mirabegron, a clinically implemented, selective β_3_-AR agonist, stimulates lipolysis and thermogenesis via the β_3_-AR, supporting the use of this class of drugs to increase human brown/beige mass, energy expenditure, and the release of metabolically beneficial mediators and cytokines (40). Our findings stress the importance of developing safer and more specific β_3_-AR agonists to activate human BAT and treat metabolic disease.

## Methods

### Isolation of stromal vascular cells from adipose tissue.

SN and SCLV fat were collected at autopsy from a young woman with a non-neoplastic condition that is not expected to affect BAT. Tissue was precured in collaboration with the Pathology Core at the National Cancer Institute at the NIH. Tissue was collected within 24 hours postmortem, and cells were isolated immediately upon collection. Briefly, 5–7 g of fat was finely minced into small pieces, and tissue was digested in HBSS containing 1% pen/strep and 50 μg/μL of gentamicin and 2 mg/mL of type 1 collagenase (Worthington Biomedical Corp) buffer for 1 hour at 37°C, gently swirling every 15 minutes. Collagenase digestion was stopped by adding an equal amount of HBSS medium containing 10% FBS and antibiotics. The mixture was filtered through a 250 μm mesh, and the filtrate was centrifuged at 50*g* for 10 minutes at room temperature. The floating cell layer was discharged while the infranatant was transferred to a sterile tube and centrifuged at 200*g* for 10 minutes to pellet the stromal vascular cells. Cell pellets were resuspended and treated with erythrocyte lysis buffer (MilliporeSigma) for 10 minutes at room temperature. After incubation, an equal volume of growth media (DMEM/F12 + 10% FBS + 1% antibiotics) was added; cells were filtered through a 40 μm mesh filter and repelleted with centrifugation (300*g* for 10 minutes). Cells were then resuspended in growth media and then plated at a density of 250,000 cells/cm^2^ in 6-well plates. All procedures were done under sterile conditions. On the following day, cells were washed with PBS and replenished with growth media. At 70%–80% confluence, cells were either subcultured or frozen (10% DMSO in growth media) for cryopreservation.

### Differentiation of human primary brown/beige adipocytes.

Two days before confluence, cells were incubated with basic FGF (4 ng/mL) (Bio-Techne). Differentiation (day 0) was initiated for 10 days via a serum-free complete differentiation media (DMEM/F12 medium supplemented with 1% antibiotics, 0.5 mM IBMX, 100 nM human insulin, 100 nM dexamethasone, 2 nM triiodothyronine, 10 μg/mL transferrin, 5 μM rosiglitazone, 33 μM biotin, 17 μM pantothenate, and 34 μM BMP7). After induction in the complete media, cells were maintained in DMEM/F12 with insulin (10 nM), dexamethasone (10 nM), biotin (33 μM), and pantothenate (17 μM) until mature adipocytes were used for metabolic experiments (day 20–day 22). This is a modified protocol (89). Differentiation of immortalized human brown adipocytes was performed as previously published (70, 71).

### Oil Red O staining.

Cells were washed twice with PBS, fixed with 4% paraformaldehyde for 1 hour, and stained with filtered Oil Red O solution (0.5% Oil Red O in isopropyl alcohol) for 4 hours at room temperature. Cells were then washed with PBS, and a photograph of the dish was taken.

### MitoTracker, LipidTOX, and UCP1 staining.

Preadipocytes were plated and differentiated on 4-well glass slides (Millicell EZ SLIDES, MilliporeSigma). For visualization of mitochondria, live cells were washed with warm media and incubated with MitoTracker Red CM-H2XRos (250 nM) for 20 minutes, then fixed with 4% paraformaldehyde. For staining of lipid droplets, fixed cells were incubated with HCS LipidTOX Green (1:200) for 30 minutes at room temperature after permeabilization with 0.1% Triton X-100 for 5 minutes. Subsequently, cells were incubated overnight with UCP1 (ab155117, Abcam) (1:250), washed in PBS, and incubated with the relevant secondary antibody at room temperature for 1 hour. After the final washes, antifade mounting medium with DAPI (VECTASHIELD HardSet, Vector Laboratories, Maravai LifeSciences) was added to the slides, and images were taken the following day. Image acquisition was performed with an upright Zeiss Axio Observer Z1 microscope using Zen software (2012; Zeiss). Images were minimally processed to adjust brightness and contrast.

### RNA extraction and gene expression.

Total RNA was extracted using TRIzol (Invitrogen, Thermo Fisher Scientific) following the manufacturer’s protocol. RNA quantity and quality were determined by spectrophotometry (NanoDrop, Thermo Fisher Scientific). A total of 1 μg of total RNA was reverse-transcribed using High Capacity cDNA Reverse Transcription kit (Applied Biosystems, Thermo Fisher Scientific). Quantitative reverse transcriptase PCR was run in duplicates using SYBR green fluorescent dye (Bio Basic) and quantified in the ABI PRISM 7900HT Sequence Detection System (Applied Biosystems, Thermo Fisher Scientific). Relative mRNA expression was determined by the Δ-Ct method using TATA-binding protein as an endogenous housekeeping control. All sequences of primers used in this study are provided in Supplemental Table 1. All gene expression data are expressed on a log_10_ scale. Ct values of all genes analyzed are provided in Supplemental Tables 2–4. When comparing gene expression between undifferentiated and differentiated adipocytes, gene expression was normalized to the undifferentiated cells. When comparing mRNA expression between siRNA-Ctrl and siRNA-*ADRB1* or siRNA-*ADRB3*, gene expression was normalized to siRNA-Ctrl adipocytes.

### Western analysis.

Preadipocytes or differentiated adipocytes were washed twice with ice-cold PBS and lysed in RIPA buffer (50 mM Tris pH 7.4; 150 mM NaCl; 1 mM EDTA; 1% Triton X-100; 0.1% SDS) containing cOmplete Protease Inhibitor Cocktail (Roche). Cell lysates were sonicated and clarified by centrifugation at 14,000*g* for 20 minutes at 4°C. Protein concentrations of cell supernatants were determined using the bicinchoninic acid reagents (Pierce, Thermo Fisher Scientific) using BSA as the standard. Proteins (20 μg) were separated by SDS-PAGE (Bio-Rad) and transferred to PVDF membranes (Bio-Rad). Membranes were blocked in Tris-buffered saline (pH 7.5) containing 0.05% Tween 20 and 5% milk for 1 hour at room temperature and then probed with primary antibodies produced in rabbit specific to FABP4 (2120, Cell Signaling Technology, 1:1000), adiponectin (2789, Cell Signaling Technology, 1:1000), UCP1 (ab155117, Abcam, 1:500), HSL660 (4126, Cell Signaling Technology, 1:1000), HSL563 (4139, Cell Signaling Technology, 1:1000), HSL (4107, Cell Signaling Technology, 1:1000), ATGL (2439, Cell Signaling Technology, 1:1000), Adhb5/CGI-58 (ab183739, Abcam, 1:1000), perilipin-1 (9349, Cell Signaling Technology, 1:1000), p-(Ser/Thr) PKA substrate (9621, Cell Signaling Technology, 1:1000), OxPhos Rodent WB Antibody Cocktail (45-8099, Invitrogen, Thermo Fisher Scientific, 1:1000), Tom20 (42406, Cell Signaling Technology, 1:1000), and β-actin (A2228, MilliporeSigma, 1:5000) overnight at 4°C. Bound antibodies were detected with horseradish peroxidase–conjugated linked anti-rabbit (sc-2004, Cell Signaling Technology, 1:10,000) or anti-mouse (7076P2, Cell Signaling Technology, 1:10,000) secondary antibodies and visualized by enhanced chemiluminescence (Bio-Rad).

### Knockdown of ADRB1 and ADRB3 by RNA interference.

For RNA interference–mediated gene *ADRB1* and *ADRB3* knockdown, siGENOME SMARTpool Human *ADRB1* (M-005425-02-0020) and siGENOME SMARTpool Human *ADRB3* (M-005427-00-0020) were obtained from GE-Dharmacon. Nontargeting siRNAs (D-001206 GE-Dharmacon) were used as the control. To reduce levels of endogenous *ADRB3* and *ADRB1*, we transfected differentiated brown/beige adipocytes from stroma vascular fraction of SCLV fat and immortalized brown adipocytes with siRNA-*ADRB3* or siRNA-Ctrl (20 pmol) using Lipofectamine RNAiMAX reagent following the manufacturer’s protocol (Thermo Fisher Scientific). On the day of transfection, mature adipocytes were incubated in OptiMEM (Gibco, Thermo Fisher Scientific) supplemented with siRNA/Lipofectamine reagent, supplemented with only 1% antibiotics. After 48 hours or 72 hours of transfection, cells were either harvested for gene expression to validate knockdown of *ADRB1* and *ADRB3* or used for functional analysis including lipolysis and cellular respiration.

### Lipolysis assay.

Preadipocytes were plated and differentiated on 12-well plates (3512, Corning). The day prior to the assay, differentiated immortalized and primary cells not treated with siRNA were switched from the maintenance media to serum- and hormone-free DMEM or DMEM/F12 with 1% antibiotic medium overnight. Treatments were done in Krebs-Ringer bicarbonate (KRB) buffer with 4% fatty acid–free BSA and 5 mM glucose with adenosine deaminase (ADA) (1 U/mL) and PIA (20 nM) to standardize potential variations in adenosine levels (90). Lipolysis was stimulated under basal and stimulated conditions for 45 minutes using Fsk (an AC activator), Iso (a pan–β-AR agonist), dobutamine (β_1_-AR agonist), terbutaline (β_2_-AR agonist), or mirabegron (β_3_-AR agonist). For experiments with β-AR antagonists, we pretreated the adipocytes for 1 hour with propranolol (a pan–β-AR agonist) or L-748,337 (β_3_-AR antagonist) and then stimulated the cells with the β-AR agonists. Lipolysis was assessed from the release of glycerol in the incubation medium as previously described (91), using free glycerol reagent (MilliporeSigma). All reagents, except mirabegron (Bio-Techne), were purchased from MilliporeSigma.

### Determination of cAMP concentration in differentiated adipocytes.

To measure cAMP levels, cells were incubated in serum- and hormone-free DMEM/F12 medium overnight and subjected to 1-hour treatment with 0.250 mM IBMX, followed by exposure to 10 μM Iso or 10 μM Fsk for 45 minutes in KRB buffer containing 4% BSA, ADA, and PIA. Cells were then washed twice with cold PBS and immediately lysed, and the cellular cAMP levels were measured using an enzyme immunoassay kit (Bio-Techne).

### Cellular respiration assays.

Preadipocytes grown to 90% confluence from a 10 cm cell culture dish were plated into individual wells of XF96 cell culture microplates (Agilent Cell Analysis Technology). Once confluent, the population of cells in each were differentiated into adipocytes. After differentiation and transfection, real-time OCR and ECAR were assessed using the Seahorse XFe Extracellular Flux Analyzer (Seahorse Bioscience).

For the mitochondrial stress assay, on the day of the experiment, primary adipocytes were washed in prewarmed XF assay media supplemented with sodium pyruvate (1 mM), l-glutamine (2 mM), and glucose (25 mM) and adjusted to pH 7.4. Cells were then maintained in the same assay buffer in a non-CO_2_ incubator for 1 hour. Cellular respiration was analyzed by using the following perturbation drugs: oligomycin (2 μM), FCCP (2 μM), and respiratory chain inhibitors rotenone (0.11 μM), and antimycin A (2.2 μM). For β-AR agonists and Fsk-induced OCR, cells were first injected with the drugs mirabegron (1 nM to 10 μM) or Fsk (10 μM) or dobutamine (10 μM) or terbutaline (10 μM) followed by oligomycin, FCCP, and rotenone and antimycin A at the same concentration reported above. All lipolytic agonist-stimulated OCR experiments were performed in the presence of 2% BSA.

For the glucose oxidation assay, primary adipocytes were washed in prewarmed XF assay media only adjusted to pH 7.4, with no exogenous supplements added. Cells were then maintained in the same assay buffer in a non-CO_2_ incubator for 1 hour. Cellular respiration was analyzed by using the following perturbation drugs: glucose (25 mM) followed by oligomycin, FCCP, and rotenone and antimycin A at the same concentration reported above.

For the glycolysis assay, primary adipocytes were washed in prewarmed XF assay media supplemented with 2 mM l-glutamine only adjusted to pH 7.4. Cells were then maintained in the same assay buffer in a non-CO_2_ incubator for 1 hour. Cellular respiration was analyzed by using the following perturbation drugs: glucose (25 mM) followed by oligomycin (2 μM) and 2-deoxyglucose (100 mM).

All drugs were loaded together into the injection ports in the XFe-96 sensor cartridge, and the XF96 analyzer was operated under the manufacturer’s basal protocol at 37°C. Measurements were normalized by protein content (BSA assay). Nonmitochondrial respiration was subtracted from basal, uncoupled, and FCCP respiration, a standard calculation adjustment that is part of the Seahorse XF Cell Mito Stress Test Report Generator. Data were then normalized to basal OCR of each transfection treatment. The data set was analyzed by XFe-96 software and GraphPad Prism Software, and energy plots were generated by following the manufacturer’s guidelines and instructions (Seahorse, Inc).

### Statistics.

Data are presented as means ± SEM. Significance between groups was determined using 2-tailed unpaired Student’s *t* test, or 1-way or 2-way ANOVA when appropriate, with multiple comparisons using Bonferroni’s correction. *P* ≤ 0.05 was considered statistically significant. All statistical analysis was performed using Prism software version 8 (GraphPad).

### Study approval.

This study followed the institutional guidelines of and was approved by the Human Studies Institutional Review Boards of Beth Israel Deaconess Medical Center (Boston, Massachusetts, USA) and Joslin Diabetes Center. Written informed consent was obtained and documented (71).

## Author contributions

CC and AMC designed the experiments. CC and AMC wrote the manuscript. CC prepared primary brown/beige adipocytes and performed all the experiments. KYZ and HJL differentiated the immortalized brown adipocytes and performed Western blotting. FS and YHT provided technical assistance with experiments on the immortalized adipocytes. All authors reviewed, edited, and approved the final manuscript.

## Supplementary Material

Supplemental data

## Version 1 06/08/2021

Electronic publication
