# Supplementary material for: β3-Adrenergic receptors regulate human brown/beige adipocyte lipolysis and thermogenesis
Source: JCI Insight. 2021 Jun 8;6(11):e139160. doi: 10.1172/jci.insight.139160 (PMC8262278; doi:10.1172/jci.insight.139160)
Supplement: Supplemental data [file jciinsight-6-139160-s035.pdf]

Supplemental Information

β3-adrenergic receptors regulate human brown/beige adipocyte lipolysis and thermogenesis

Cheryl Cero [1], Hannah J. Lea [1], Kenneth Y. Zhu [1], Farnaz Shamsi [2], Yu-Hua Tseng [2], Aaron M. Cypess[1,*]

[1]Diabetes, Endocrinology, and Obesity Branch, NIDDK, NIH, Bethesda, MD
[2]Section on Integrative Physiology and Metabolism, Joslin Diabetes Center, Harvard Medical School, Boston, MA

*Correspondence: aaron.cypess@nih.gov

Contents: Supplemental Figures = 7; Supplemental Tables = 4

Supplemental Figures Titles and Legends

Supplemental Figure 1. Genetic and phenotypic profile of human supraclavicular primary pre-and differentiated adipocytes. (A-C) Gene and protein expression of UCP1 in superficial neck (WAT) and supraclavicular (BAT) adipose tissue (n=1) (A); gene expression of brown/beige adipocyte markers (B) and adrenergic receptors (C) in WAT and BAT tissues. (D-F) Differentiation protocol and differentiation progression of primary human brown/beige adipocytes (D); gene expression of *PPARGC1A*, *UCP1* and *MT-CO2* (E) and protein expression of UCP1, TOM20 and Actin (F) over differentiation. (G-J) qPCR of late markers of differentiation (G); brown/beige adipocyte markers (H); fatty acid oxidation genes (I); and beta-adrenergic receptors (J) in pre- and mature primary supraclavicular adipocytes. Gene expression data are normalized to pre-adipocytes and expressed as mean ± SEM on a Log 10 scale. Data are analyzed by 2-tailed unpaired Student's t-test and a One-way ANOVA with *P<0.05, **P<0.01, ***P<0.001, ****P<0.0001.

Supplemental Figure 2. Silencing *ADRB3* in mature brown/beige adipocytes lowers UCP1 protein content but does not compromise lipid accumulation. (A-C) Immunofluorescence of differentiated adipocytes staining for mitochondria (Mitotracker, red), lipid droplets (LipidTox-green), anti-UCP1 (white) and nuclei (Dapi, blu) in untreated siRNA (A); siRNA-Ctrl (B) and siRNA-*ADRB3* (C) adipocytes. Scale bars: 100 µm for all images. (D) Quantification of LipidTox positive adipocytes in untreated, siRNA-Ctrl and siRNA-*ADRB3* adipocytes. Data are the mean of five sections analyzed by One-way ANOVA.

Supplemental Figure 3. Silencing *ADRB3* in mature brown/beige adipocytes does not compromise beige/brown adipocyte identity and ETC genes and proteins. (A) qRT-PCR analysis of human brown/beige adipocyte markers (B-C) mRNA expression of nuclear encoded electron transport chain genes (B) and proteins (C) in siRNA Ctrl and siRNA-*ADRB3* adipocytes. Gene expression data are normalized to siRNA-Ctrl adipocytes and expressed as mean ± SEM on a Log 10 scale. Data are analyzed by 2-tailed unpaired Student's t-test with *P<0.05, **P<0.01, ***P<0.001, ****P<0.0001.

Supplemental Figure 4. Activation of all three beta-adrenergic subtypes in primary human brown/beige adipocytes stimulates lipolysis and can be suppressed by

pre-treatment of the β_3-AR antagonist L-748,337. (A) Basal, beta-agonists, and iso (10 µM) stimulated lipolysis with and without pre-treatment of propranolol (10 µM) and β3-AR antagonist L-748,337 (10 µM). Data are represented as mean ± SEM. 2-way ANOVA was used for statistical analysis. * when comparing basal to beta agonist stimulated lipolysis; # when comparing beta-agonist stimulation to propranolol; $ when comparing beta-agonist stimulation to L-748,337 pre-treatment. *P<0.05; **P<0.01; ***P<0.001; ****P<0.0001.

Supplemental Figure 5. Silencing *ADRB3* in mature brown/beige adipocytes lowers non-mitochondrial respiration. (A-B) Non-mitochondrial respiration of differentiated siRNA-Ctrl and siRNA-*ADRB3* transfected adipocytes without ligand stimulated OCR (A) and Fsk-stimulated OCR (B). (C) mRNA expression of human genes encoding and glucose transporters 1 (*SLC2A1*) and 4 (*SLC2A4*) in transfected adipocytes. Gene expression data are normalized to siRNA-Ctrl adipocytes and expressed as mean ± SEM on a Log 10 scale.

Supplemental Figure 6. Phenotype of immortalized human brown/beige adipocytes. (A) Representative microscopic pictures of the morphology of undifferentiated and differentiated immortalized brown adipocytes. (B) Oil Red O staining of differentiated adipocytes at passage number 18 (P18). (C-D) qPCR of late markers of differentiation (C); brown/beige adipocyte markers in pre- and mature adipocytes (D). (E) Protein expression of FABP4, adiponectin and UCP1 at P18 in pre- and mature adipocytes. (F-G) Gene expression of the mitochondrial encoded gene MT-CO2 (F); beta-adrenergic receptors (G) in undifferentiated and differentiated immortalized brown adipocytes. (H) Gene expression of beta-adrenergic receptors and UCP1 in pre-adipocytes and mature adipocytes of primary and immortalized brown/beige adipocytes. PCR data are normalized to pre-adipocytes and expressed as mean ± SEM on a Log 10 scale, except for data in (H) that is not normalized. Data are analyzed by 2-tailed unpaired Student's t-test. *P<0.05; **P<0.01; ***P<0.001; ****P<0.0001.

Supplemental Figure 7. Silencing *ADRB3* in immortalized human brown adipocytes lowers the cellular lipogenic and thermogenic machinery. (A-G) mRNA expression profiles of beta-adrenergic receptor genes (A); thermogenic related genes (B); fatty acid synthesis genes (C); fatty acid oxidation genes (D); mtDNA encoded

genes (E); glucose transporters (F); and lipogenic genes (G) in 48h transfected siRNA-Ctrl and siRNA-*ADRB3* immortalized adipocytes. (H-I) Basal and Fsk (10 μM) stimulated cAMP concentration (H) and western blot of PKA substrates in basal and Fsk 10 μM stimulated transfected adipocytes (I). (J-K) Basal and Fsk (10 μM) stimulated glycerol release (J) and protein expression of pHSL563; pHSL660; HSL; ATGL and actin in siRNA-Ctrl and siRNA siRNA-*ADRB3* adipocytes following treatment. (L-N) OCR by differentiated cells treated in immortalized siRNA-Ctrl (black); siRNA-*ADRB1* (gray) and siRNA-*ADRB3* (red) transfected adipocytes (L); quantification of basal respiration (M) and metabolic profile (N) by transfected immortalized adipocytes. Data are expressed as mean ± SEM. Gene expression data are normalized to siRNA-Ctrl adipocytes, expressed on a Log 10 scale and analyzed by 2-tailed unpaired Student's t-test. OCR data are analyzed by one-way ANOVA. cAMP and lipolysis data are analyzed by 2-way ANOVA with * when comparing basal vs stimulated in siRNA-Ctrl; $ when comparing basal to stimulated in siRNA-*ADRB3* adipocytes; # when comparing same doses between siRNA-Ctrl and siRNA-*ADRB3* adipocytes. *P<0.05; **P<0.01; ***P<0.001; ****P<0.0001.

Supplemental Table 1. List of genes and sequences of the primers used for RT-PCR gene expression analysis.

Supplemental Table 2. Ct values of genes investigated in the main figures 1, 2 and 5.

Supplemental Table 3. Ct values of genes investigated in the supplemental figure 1.

Supplemental Table 3. Ct values of genes investigated in the supplemental figures 2,4 and 5.

Supplemental Figure 1. Genetic and phenotypic profile of human supraclavicular primary pre-and differentiated adipocytes.

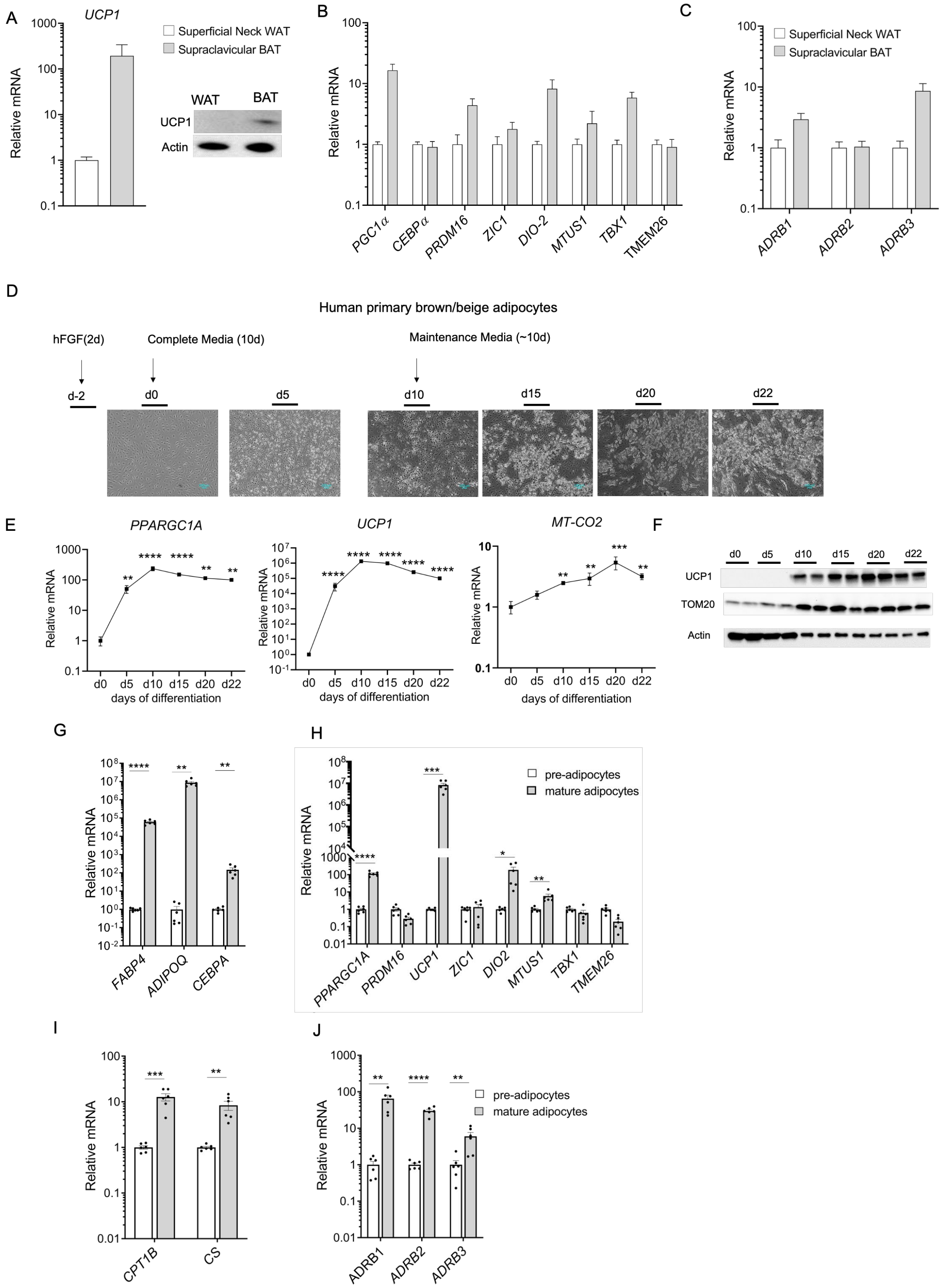

Supplemental Figure 2. Silencing *ADRB3* in mature brown/beige adipocytes lowers UCP1 protein content but does not compromise lipid accumulation.

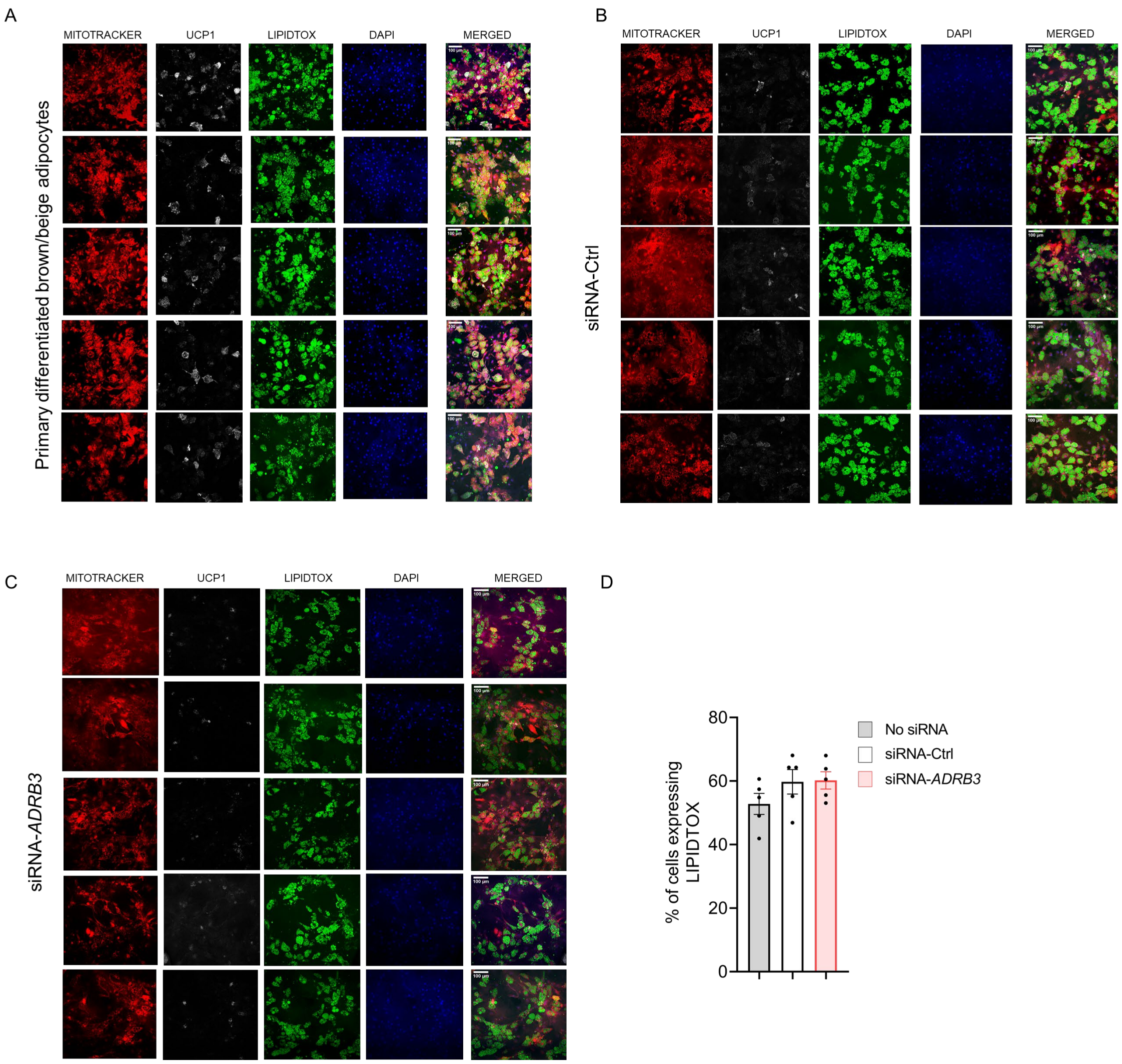

Supplemental Figure 3. Silencing *ADRB3* in mature brown/beige adipocytes does not compromise beige/brown adipocyte identity and ETC genes and proteins.

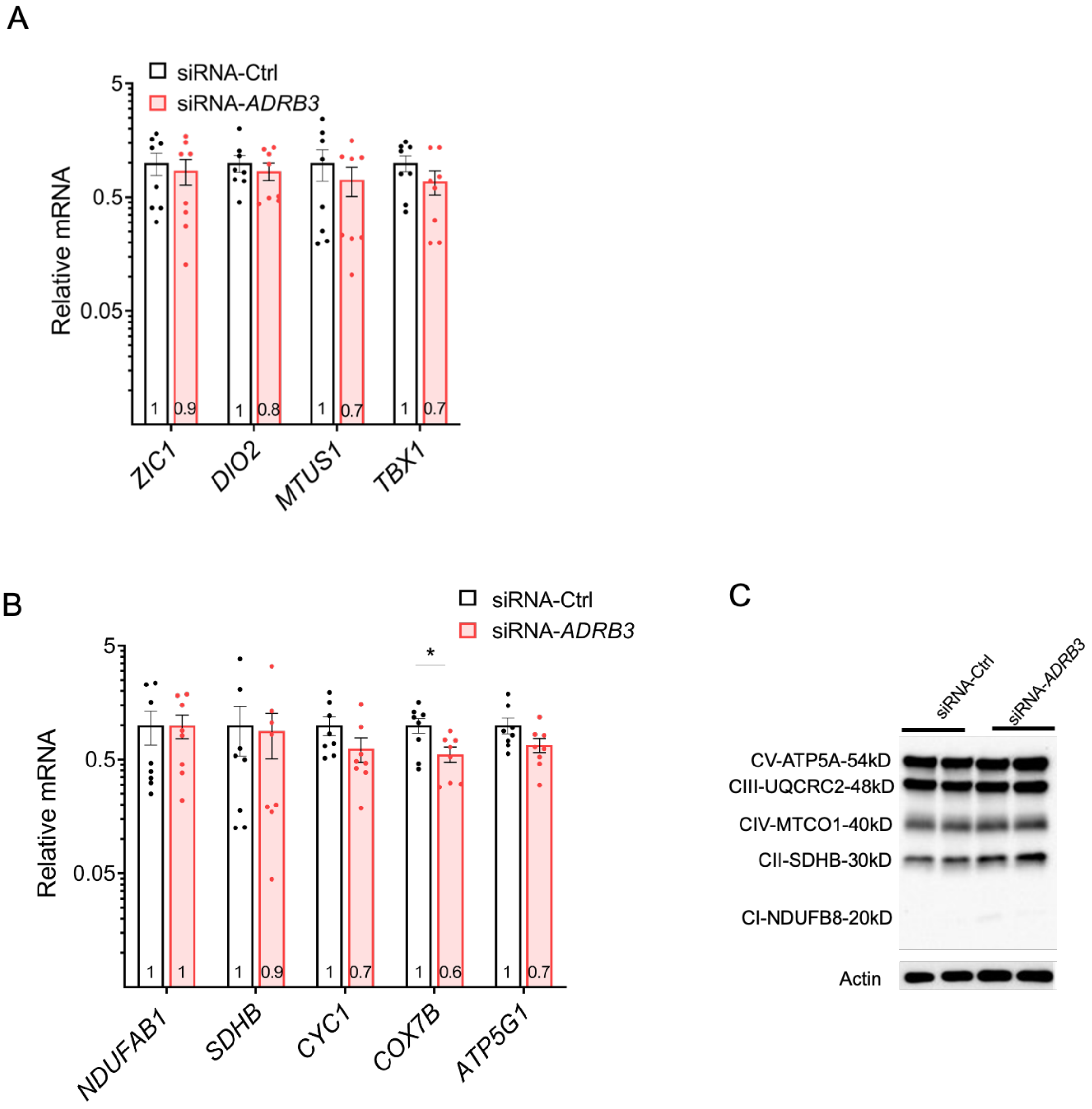

Supplemental Figure 4. Activation of all three beta-adrenergic subtypes in primary human brown/beige adipocytes stimulates lipolysis and can be suppressed by pre-treatment of the β_3-AR antagonist L-748,337.

A

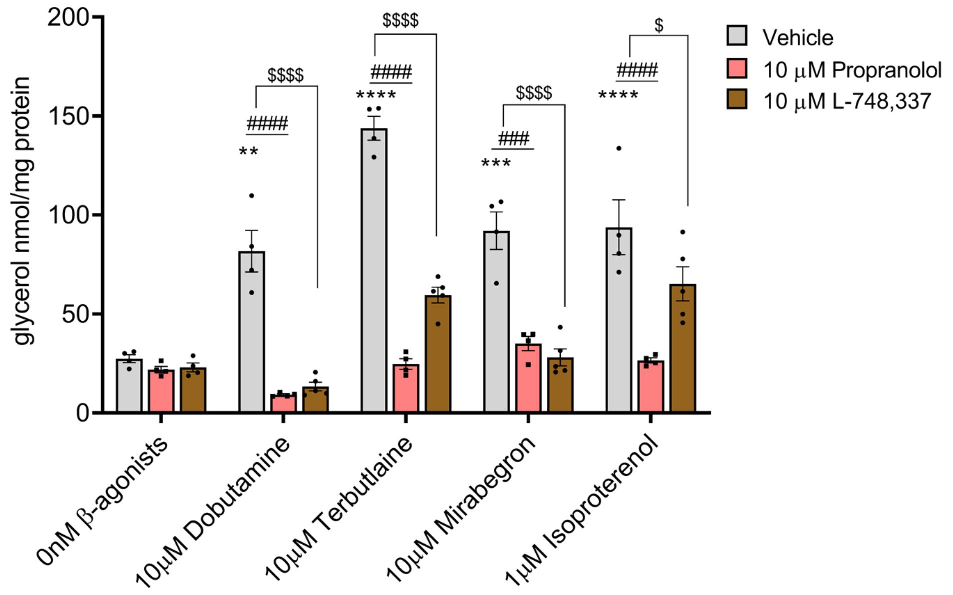

Supplemental Figure 5. Silencing *ADRB3* in mature brown/beige adipocytes lowers non-mitochondrial respiration.

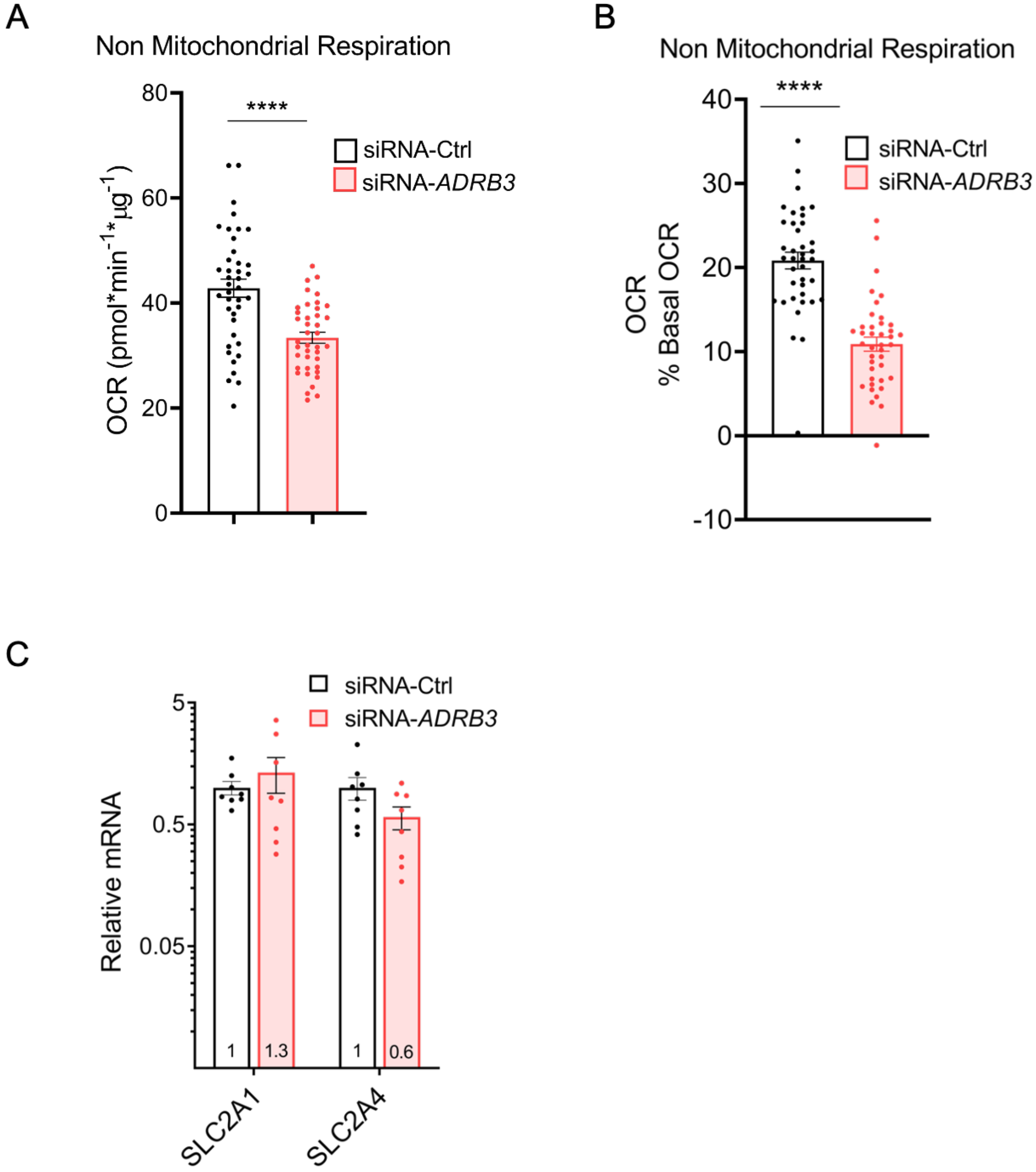

Supplemental Figure 6. Phenotype of immortalized human brown/beige adipocytes.

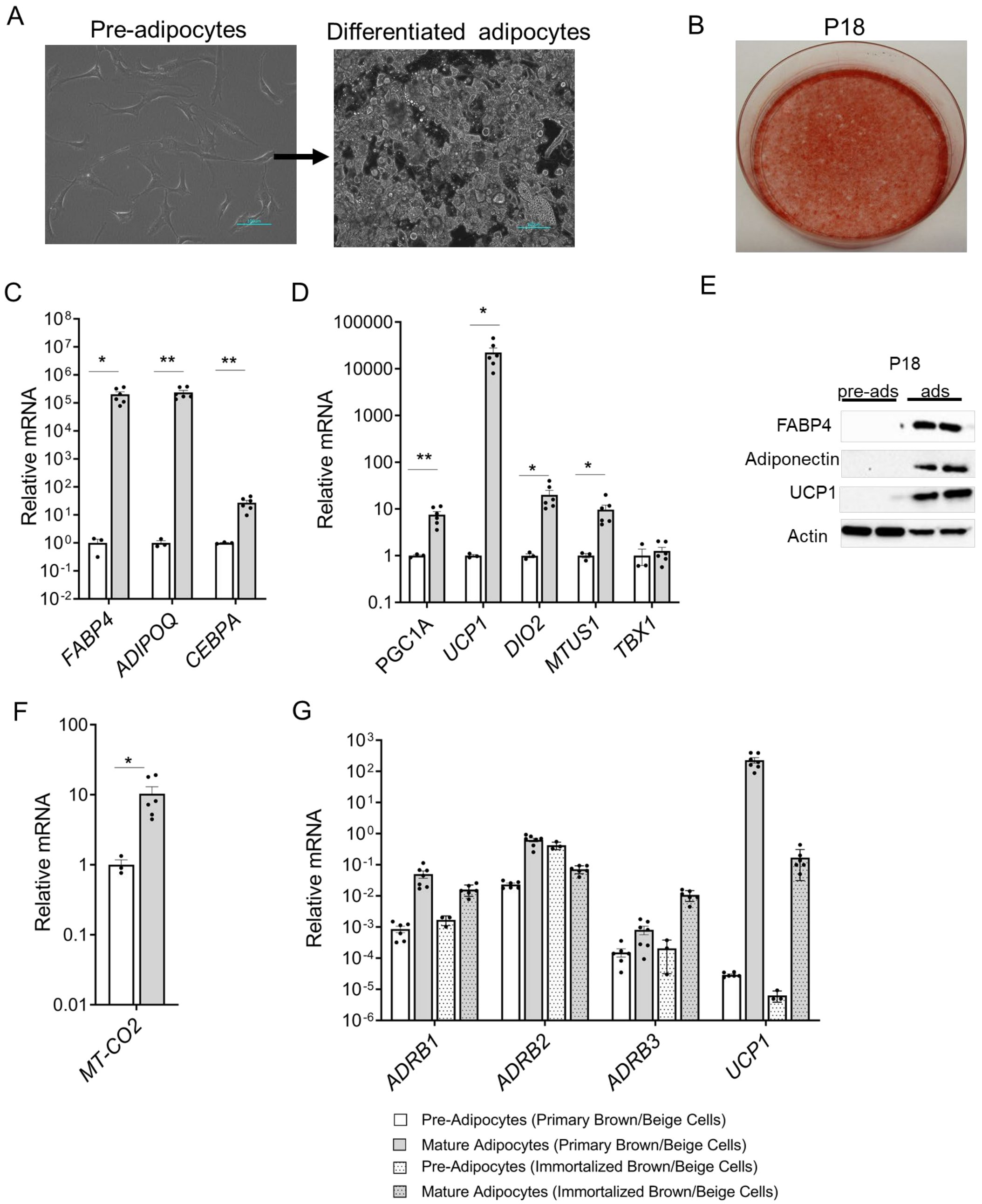

Supplemental Figure 7. Silencing *ADRB3* in immortalized human brown adipocytes lowers the cellular lipogenic and thermogenic machinery.

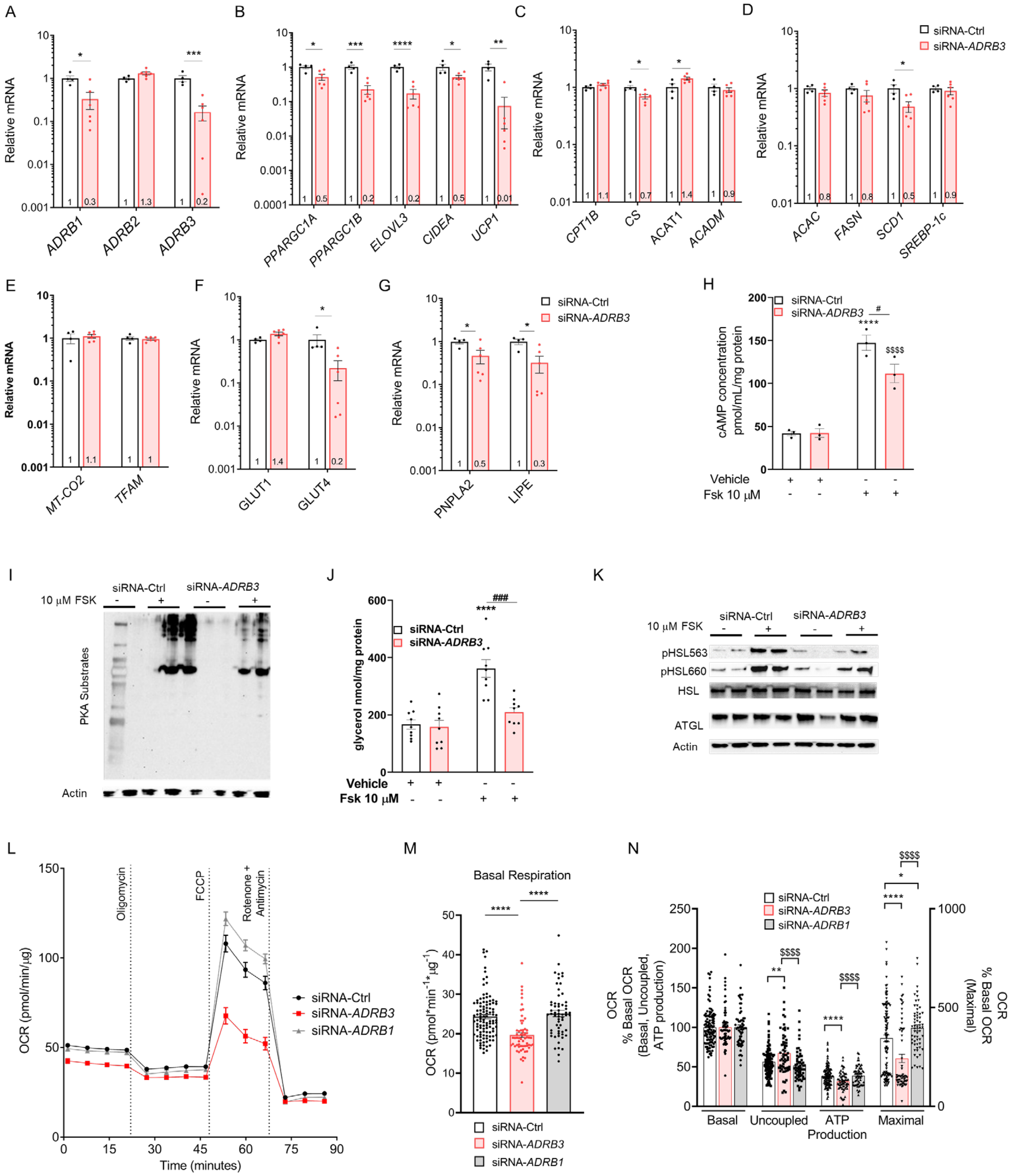

Supplemental Table 1. List of genes and sequences of the primers used for RT-PCR gene expression analysis.

Gene	Prime	Primer Sequence
TBP	sense	5'-CCC GAA ACG CCG AAT ATA A-3'
	antisense	**5'-GAA AAT CAG TGC CGT GGT TC-3'**
ACTIN	sense	5'-GAG CTA CGA GCT GCC TGA CG-3'
	antisense	**5'-GTA GTT TCG TGG ATG CCA CAG-3'**
ADIPOQ	sense	5'-CCT GGT GAG AAG GGT GAG AA-3'
	antisense	**5'-GTA AAG CGA ATG GGC ATG TT-3'**
FABP4:	sense	5'-CCT TTA AAA ATA CTG AGA TTT CCT TCA-3'
	antisense	**5'-GGA CAC CCC CAT CTA AGG T-3'**
PPARGC1A	sense	5'-CCA CAG AGA ACA GAA ACA GCA-3'
	antisense	**5'-TGG GGT CAG AGG AAG AGA TAA-3'**
PPARGC1B	sense	5'-GAT GCC AGC GAC GAC TTT GAC TC-3'
	antisense	**5'-ACC CAC GTC ATC TTC AGG GA-3'**
PNPLA2	sense	5'-ACC AGC ATC CAG TTC AAC CT-3'
	antisense	**5'-ATC CCTGCTTGC ACA TCT CT-3'**
LIPE	sense	5'-CTC AGT GTG CTC TCC AAG TG-3'
	antisense	**5'-CAC CCA GGC GGA AGT CTC-3'**
ABHD5	sense	5'-GAG GTT TTT CTT AGG TGC TGG-3'
	antisense	**5'-TGT CAG GGT GCA TTT TAC CA-3'**
CEBPA	sense	5'-AAG AAG TCG GTG GAC AAG AAC-3'
	antisense	**5'-GTC ATT GTC ACT GGT CAG CTC-3'**
UCP1	sense	5'-CCA ACT GTG CAA TGA AAG TGT-3'
	antisense	**5'-CAA GTC GCA AGA AGG AAG GTA-3'**
MT-CO2	sense	5'-AGT CCT GTA TGC CCT TTT CCT-3'
	antisense	**5'-TTC AGA CGG TTT CTA TTT CCT G-3'**
CPT1B	sense	5'-CTC CTT TCC TTG CTG AGG TG-3'
	antisense	**5'-TCT CGC CTG CAA TCA TGT AG-3'**
CS	sense	5'-GGA CAT ATC CCA ACA GAG GAA-3'
	antisense	**5'-CTC TTT GCC CAC TCT TTT GAG-3'**
ADRB1	sense	5'-CTC CTT CTT CTG CGA GCT GT-3'
	antisense	**5'- AGG ATG GGC AGG AAG GAC-3'**
ADRB2	sense	5'-CTT CTT GCC CAT TCA GAT GC-3'
	antisense	**5'-AAG AAG TCA CAG CAG GTC TC-3'**
ADRB3	sense	5'-TTT GCC AAC GGC TCG AC-3'
	antisense	**5'-CGT CAG GTT CTG GAG GGT AG-3'**
ZIC1	sense	5'-CAA ACA CAT GAA GGT CCA CG-3'
	antisense	**5'-ATA AGG AGC TTG TGG TCG G-3'**
DIO-2	sense	5'-CCT CCT CGA TGC CTA CAA AC-3'
	antisense	**5'-GCT GGC AAA GTC AAG AAG GT-3'**
MTUS1	sense	5'-TGA GGC AAA TAG CTG CTC CA-3'
	antisense	**5'-TGA GGA GAT ACG GCT CGA TCA-3'**
TMEM26	sense	5'-ATG GAG GGA CTG GTC TTC CTT-3'
	antisense	**5'-CTT CAC CTC GGT CAC TCG C-3'**
TBX1	sense	5'-ACG ACA ACG GCC ACA TTA TTC-3'
	antisense	**5'-CCT CGG CAT ATT TCT CGC TAT CT-3'**
PRDM16	sense	5'-CAC GAG TGC AAG GAC TGC-3'
	antisense	**5'-TGT GGA TGA CCA TGT GCT G-3'**
ACAT1	sense	5'-CCT GAG GAA GAT GAA GAC C-3'
	antisense	**5'-CTC TGC CTC TGC TGT CAA C-3'**
ACADM	sense	5'-TTG AGT TCA CCG AAC AGC AG-3'
	antisense	**5'-AGG GGG ACT GGA TAT TCA CC-3'**
GLUT1	sense	5'-CTG CTC ATC AAC CGC AAC-3'
	antisense	**5'-CTT CTT CTC CCG CAT CAT CT-3'**
GLUT4	sense	5'-TGG GCT TCT TCA TCT TCA CC-3'
	antisense	**5'-GTG CTG GGT TTC ACC TCC T-3'**
NDUFAB1	sense	5'-CGT CTC GTC GTC CTT TCA GC-3'
	antisense	**5'-CCT GGA TGC CCT CTA ACG-3'**
SDHB	sense	5'-GGA GAG CGA CCT CGG GGT T-3'
	antisense	**5'-GTC TCT GTG GCT TTC CTG ACT-3'**
CYC1	sense	5'-GTC GTC GAA GTC TGG CCT TT-3'
	antisense	**5'-CAC GGT GAG ACC ACG GAT AG-3'**
COX7B	sense	5'-AGC GAA TTG GCA CCA AAG CAG CA-3'
	antisense	**5'-CTG GTG GCT CTG CCT TGC CAT-3'**
ATP5G1	sense	5'-CTA AAC AGC CTT CCT ACA GCA ACT T-3'
	antisense	**5'-TGA ACC AGC CAC ACC AAC TGT-3'**
ACAC	sense	5'-GAG AAC TGC CCT TTC TGC AC-3'
	antisense	**5'-CCA AGC TCC AGG CTT CAT AG-3'**
FASN	sense	5'-ACT GGA GGG GAC AGT GCA T-3'
	antisense	**5'-GCA GCG CTG TTT ACA CTC CT-3'**
SCD1	sense	5'-CAC CAC ATT CTT CAT TGA TTG CA-3'
	antisense	**5'-ATG GCG GCC TTG GAG ACT-3'**
SREBP-1c	sense	5'-GGC TCC TGC CTA CAG CTT CT-3'
	antisense	**5'-CAG CCA GTG GAT CAC CAC A-3'**
PGC1B	sense	5'- GAT GCC AGC GAC GAC TTT GAC TC-3'
	antisense	**5'-ACC CAC GTC ATC TTC AGG GA-3'**
ELOVL3	sense	5'- CTG TTC CAG CCC TAT AAC TTC G-3'
	antisense	**5'-GAA TGA GGT TGC CCA ATA CTC C-3'**
CIDEA	sense	5'- ACT CTG GAT GCC CTC GTC AT-3'
	antisense	**5'-ACT CTT CTG TGT CCA CCA CG-3'**
TFAM	sense	5'-AGC TCA GAA CCC AGA TGC-3'
	antisense	**5'-CCA CTC CGC CCT ATA AGC-3'**

Supplemental Table 2. Ct values of genes investigated in the main figures 1, 2 and 5.

CT Values of Genes Figure 1				
Gene	siRNA-Ctrl		siRNA-ADRB3	
	average	st.dev	average	st.dev
TBP	24.6	± 0.3	24.4	± 0.2
ADRB1	26.4	± 0.7	28.2	± 0.9
ADRB2	27.4	± 0.24	27.7	± 0.3
ADRB3	29.7	± 0.4	35.1	± 1.0
PGC1A	24.1	± 0.5	24.8	± 0.2
PGC1B	25.3	± 0.4	26.2	± 0.3
ELOVL3	24.7	± 0.7	26.0	± 0.5
CIDEA	27.0	± 0.1	27.3	± 0.2
UCP1	21.4	± 0.7	24.4	± 0.5
MT-CO2	13.4	± 0.3	13.7	± 0.1
TFAM	22.5	± 0.3	23.5	± 0.2
CPT1B	22.0	± 0.5	23.4	± 0.3
CS	20.7	± 0.3	21.1	± 0.2
ACAT1	21.6	± 0.3	22.0	± 0.4
ACADM	21.9	± 0.4	22.2	± 0.3
ACAC	21.4	± 0.3	24.4	± 0.2
FASN	21.4	± 0.5	24.4	± 0.4
SCD1	21.4	± 0.3	24.4	± 0.3
SERBP1C	21.4	± 0.4	24.4	± 0.3
CMKT1	22.5	± 0.2	23.3	± 0.2
CMKT2	24.1	± 0.4	25.0	± 0.4

CT Values of Genes expressed in Figure 2				
**Gene**	**siRNA-Ctrl**		**siRNA-ADRB3**	
	average	st.dev	average	st.dev
PNPLA2	17.7	± 0.5	17.9	± 0.4
LIPE	19.8	± 0.4	20.1	± 0.3
ABHD5	24.8	± 0.4	25.3	± 0.4

CT Values of Genes Figure 5				
Gene	siRNA-Ctrl		siRNA-ADRB1	
	average	st.dev	average	st.dev
TBP	24.0	± 0.2	24.2	± 0.1
ADRB1	24.5	± 0.1	28.8	± 0.2
ADRB2	27.2	± 0.5	27.3	± 0.2
ADRB3	29.1	± 0.5	30.8	± 0.5
PGC1A	22.9	± 0.1	23.3	± 0.1
PGC1B	24.3	± 0.1	24.7	± 0.1
ELOVL3	23.0	± 0.4	24.0	0.1
CIDEA	26.9	± 0.1	27.5	± 0.2
UCP1	19.8	± 0.3	22.5	± 0.5
MT-CO2	12.7	± 0.3	12.9	± 0.2
TFAM	22.0	± 0.2	22.4	± 0.2
CPT1B	20.9	± 0.2	22.2	± 0.2
CS	20.1	± 0.1	20.6	± 0.2
ACAT1	20.90138	± 0.1	21.0735	0.2
ACADM	20.87487	± 0.01	21.06175	0.2
ACAC	20.5	± 0.2	21.9	± 0.2
FASN	18.4	± 0.2	19.5	± 0.2
SCD1	14.0	± 0.2	14.9	± 0.2
SERBP1C	20.3	± 0.1	21.1	± 0.3
GLUT1	21.5	± 0.2	21.5	± 0.3
GLUT4	21.3	± 0.2	22.9	± 0.4
PNPLA2	16.4	± 0.2	17.2	± 0.1
LIPE	18.8	± 0.04	19.6	± 0.2
ABHD5	24.1	± 0.2	24.4	± 0.4
NDUFAB1	21.5	± 0.8	21.9	± 1
SDHB	31.0	± 0.6	30.5	± 0.5
CYC1	21.8	± 0.3	22.7	± 0.3
COX7B	18.3	± 0.2	18.7	± 0.2
ATP5G1	22.6	± 0.1	23.5	± 0.3

Supplemental Table 3. Ct values of genes investigated in the supplemental figure 1.

CT Values of Genes Extended Figure 1 (Tissue)				
Gene	Superficial neck fat		supraclavicular fat	
	average	st.dev	average	st.dev
ACTIN	20.0	± 0.1	21.2	± 0.03
UCP1	41.0	± 0	35.4	± 1
ADRB1	30.5	± 0.3	30.4	± 0.03
ADRB2	26.4	± 0.1	27.8	± 0.06
ADRB3	31.8	± 0.2	29.9	± 0.0658
PGC1A	29.8	± 0.1	27.3	± 0.02
CEBPA	29.1	± 0.1	30.8	± 0.06
PRDM16	28.6	± 0.4	27.9	± 0.01
ZIC1	29.6	± 0.8	30.2	± 0.02
MTUS1	28.5	± 0.1	29.1	± 0.6
TBX1	28.1	± 0.01	27.0	± 0.06
TMEM26	28.5	± 0.6	30.7	± 0.09

CT Values of Genes Extended Figure 1 (Differentiation Process of SCLV derived brown/beige primary adipocytes)												
Gene	d0		d5		d10		d15		d20		d22	
	average	st.dev	average	st.dev	average	st.dev	average	st.dev	average	st.dev	average	st.dev
TBP	25.1	± 0.1	25.0	± 0.05	24.3	± 0.1	24.2	± 0.1	25.2	± 0.2	25.1	± 0.2
PGC1A	30.2	± 0.5	24.5	± 0.5	21.3	± 0.2	21.8	± 0.2	23.2	± 0.3	23.3	± 0.3
UCP1	41.0	± 0	26.8	± 0.9	19.9	± 0.1	20.3	± 0.3	23.2	± 0.4	24.4	± 0.3
MT-CO2	15.0	± 0.2	14.3	± 0.2	12.8	± 0.1	12.6	± 0.4	12.7	± 0.3	13.3	± 0.3

CT Values of Genes Extended Figure 1 (pre- and mature- adipocytes)				
Gene	pre-adipocytes		mature adipocytes	
TBP	25.9	± 0.1	24.6	± 0.3
FABP4	30.4	± 0.1	13.2	± 0.2
ADIPOQ	39.5	± 0.7	14.2	± 0.4
CEBPA	29.3	± 0.1	20.9	± 0.2
PGC1a	31.2	± 0.2	22.9	± 0.4
PRDM16	26.9	± 0.3	27.4	± 0.4
UCP1	41.0	± 0	16.9	± 0.3
ZIC1	31.6	± 0.4	30.4	± 1.0
DIO2	31.8	± 0.2	23.8	± 1.0
MTUS1	30.8	± 0.1	27.1	± 0.5
TBX1	25.6	± 0.1	25.5	± 0.3
TMEM26	31.3	± 0.3	32.4	± 0.7
CPT1	26.6	± 0.1	21.6	± 0.4
CS	23.1	± 0.1	18.8	± 0.5
ADRB1	36.4	± 0.4	29.2	± 0.6
ADRB2	31.4	± 0.1	25.4	± 0.3
ADRB3	38.9	± 0.5	35.4	± 0.5

Supplemental Table 4. Ct values of genes investigated in the supplemental figures 2,4 and 5.

CT Values of Genes expressed in Extended Figure 2				
Gene	siRNA-Ctrl		siRNA-ADRB3	
	average	st.dev	average	st.dev
NDUFAB1	22.4	± 0.6	22.0	± 0.4
SDHB	30.3	± 0.4	30.3	± 0.6
CYC1	22.3	± 0.3	22.9	± 0.2
COX7B	19.5	± 0.5	20.2	± 0.3
ATP5G1	23.1	± 0.2	23.5	± 0.2
ZIC1	21.4	± 0.2	24.4	± 0.4
DIO-2	24.9	± 0.2	24.9	± 0.3
MTUS1	28.2	± 0.3	28.4	± 0.4
TBX1	24.2	± 0.5	24.7	± 0.5
GLUT1	22.1	± 0.2	21.9	± 0.6
GLUT4	22.6	± 0.5	23.3	± 0.3

CT Values of Genes Extended Figure 4 (pre-mature adipocytes)				
Gene	pre-adipocytes		mature adipocytes	
TBP	23.7	± 0.39	24.5	± 0.1
FABP4	32.3	± 0.8	15.3	± 0.1
ADIPOQ	35.0	± 0.2	17.9	± 0.1
CEBPA	29.1	± 0.1	25.1	± 0.2
PGC1A	26.1	± 0.1	23.9	± 0.2
UCP1	41.0	± 0	27.4	± 0.4
DIO2	30.3	± 0.2	26.8	± 0.1
MTUS1	29.9	± 0.2	27.4	± 0.1
TBX1	35.0	± 0.6	35.3	± 0.3
CPT1	26.5	± 0.2	23.7	± 1.3
ADRB1	33.0	± 0.3	30.6	± 0.2
ADRB2	25.0	± 0.1	28.4	± 0.1
ADRB3	36.6	± 1.4	31.2	± 0.2

	CT Values of Genes Extended Figure 4H (pre-mature adipocytes)				
	Gene	pre-adipocytes		mature adipocytes	
Primary Adipocytes	***ADRB1***	36.4	± 0.4	29.2	± 0.6
	ADRB2	31.4	± 0.1	25.4	± 0.3
	ADRB3	38.9	± 0.5	35.4	± 0.5
	UCP1	41.0	± 0	16.9	± 0.3
	Gene	pre-adipocytes		mature adipocytes	
Immortalized Adipocytes	***ADRB1***	33.0	± 0.3	30.6	± 0.2
	ADRB2	25.0	± 0.1	28.4	± 0.1
	ADRB3	36.6	± 1.4	31.2	± 0.2
	UCP1	41.0	± 0	27.4	± 0.4

CT Values of Genes Extended Figure 5				
Gene	siRNA-Ctrl		siRNA-ADRB3	
	average	st.dev	average	st.dev
TBP	24.4	± 0.4	24.1	± 0.1
ADRB1	28.1	± 0.3	30.9	± 0.6
ADRB2	26.9	± 0.1	27.1	± 0.1
ADRB3	31.3	± 0.3	35.8	± 1.2
PGC1A	22.1	± 0.2	23.7	± 0.3
PGC1B	22.6	± 0.3	25.6	± 0.4
ELOVL3	23.4	± 0.1	26.8	± 0.4
CIDEA	27.2	± 0.2	28.7	± 0.2
UCP1	27.4	± 0.5	33.3	± 0.9
MT-CO2	12.6	± 0.5	12.8	± 0.2
TFAM	20.9	± 0.2	21.6	± 0.1
CPT1B	25.3	± 0.03	25.8	± 0.1
CS	19.8	± 0.2	20.9	± 0.1
ACAT1	20.9	± 0.2	20.9	± 0.1
ACADM	20.8	± 0.2	21.5	± 0.2
ACAC	20.4	± 0.2	21.3	± 0.2
FASN	20.8	± 0.2	22.0	± 0.3
SCD1	14.7	± 0.3	16.4	± 0.3
SERBP1C	22.0	± 0.1	22.8	± 0.2
GLUT1	23.5	± 0.2	23.6	± 0.2
GLUT4	23.7	± 0.5	27.6	± 0.9
PNPLA2	17.9	± 0.2	20.0	± 0.5
LIPE	21.0	± 0.3	24.0	± 0.7
